# Bayesian mixture models for phylogenetic source attribution from consensus sequences and time since infection estimates

**DOI:** 10.1177/09622802241309750

**Published:** 2025-02-12

**Authors:** Alexandra Blenkinsop, Lysandros Sofocleous, Francesco Di Lauro, Evangelia Georgia Kostaki, Ard van Sighem, Daniela Bezemer, Thijs van de Laar, Peter Reiss, Godelieve de Bree, Nikos Pantazis, Oliver Ratmann, on behalf of the HIV Transmission Elimination Amsterdam (H-TEAM) Consortium

**Affiliations:** 1 Department of Mathematics, 4615 Imperial College London, London, UK; 2 Big Data Institute, Nuffield Department of Medicine, University of Oxford, Oxford, UK; 3 Department of Hygiene, Epidemiology and Medical Statistics, Medical School, National and Kapodistrian University of Athens, Athens, Greece; 4 198321 Stichting HIV Monitoring, Amsterdam, the Netherlands; 5 Department of Donor Medicine Research, Sanquin, the Netherlands; 6 Amsterdam Institute for Global Health and Development, Amsterdam, the Netherlands; 7 Department of Global Health, Amsterdam University Medical Centers, Amsterdam, the Netherlands; 8 Division of Infectious Diseases, Department of Internal Medicine, Amsterdam Infection and Immunity Institute, Amsterdam, the Netherlands

**Keywords:** phylodynamics, HIV prevention, evolutionary clock

## Abstract

In stopping the spread of infectious diseases, pathogen genomic data can be used to reconstruct transmission events and characterize population-level sources of infection. Most approaches for identifying transmission pairs do not account for the time passing since the divergence of pathogen variants in individuals, which is problematic in viruses with high within-host evolutionary rates. This prompted us to consider possible transmission pairs in terms of phylogenetic data and additional estimates of time since infection derived from clinical biomarkers. We develop Bayesian mixture models with an evolutionary clock as a signal component and additional mixed effects or covariate random functions describing the mixing weights to classify potential pairs into likely and unlikely transmission pairs. We demonstrate that although sources cannot be identified at the individual level with certainty, even with the additional data on time elapsed, inferences into the population-level sources of transmission are possible, and more accurate than using only phylogenetic data without time since infection estimates. We apply the proposed approach to estimate age-specific sources of HIV infection in Amsterdam tranamission networks among men who have sex with men between 2010 and 2021. This study demonstrates that infection time estimates provide informative data to characterize transmission sources, and shows how phylogenetic source attribution can then be done with multi-dimensional mixture models.

## 1. Introduction

**Figure 1.**
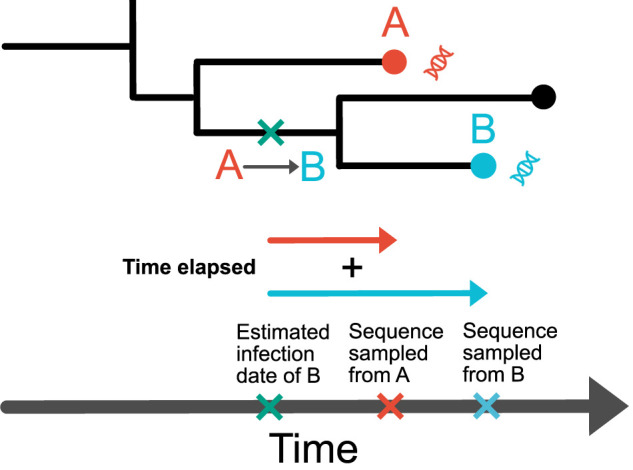
Schematic of a phylogenetic tree illustrating how to approximate time elapsed. We consider that A transmitted to B, with the sequence sampling dates of A and B known. We estimate the infection date of individual B and estimate the time elapsed as the cumulative time between the infection date of B and each of the sequence sampling dates of A and B.

Genomic surveillance of human pathogens is increasingly used to help combat the spread of infectious diseases such as COVID-19, antimicrobial-resistant bacteria, Ebola virus, or HIV.^1–5^ This involves the sequencing of the genetic code of pathogen samples obtained from diagnosed individuals^6^ or the environment such as wastewater,^7,8^ and then phylogenetic analyses are used to estimate ancestral relationships between samples based on an assumed model which describes the rate of mutation as pathogens evolve.^9^ Inferred evolutionary relationships are represented by phylogenetic trees, in which branch lengths represent the degree of genetic variation between samples that appear as tips of the tree, and these phylogenetic trees are indicative of epidemic transmission networks among human hosts.^10^ These methods can be used for example to detect new circulating pathogens or pathogen variants,^11^ determine growth rates,^12^ quantify modes of disease spread,^13^ or characterize population-level drug resistance.^14,15^ Particular interest centers on reconstructing pathogen transmission, with the primary aim to identify population-level factors that underpin disease spread.^16,17^ It is usually not possible to determine with certainty from the genetic data alone that one individual is the source of infection in another person, particularly in the case of fast-evolving pathogens such as HIV. For instance, it is common to observe genetically near-identical HIV sequences between women, even though HIV transmission between women is highly unlikely, and molecular patterns of the near-identical virus suggest instead that one or more men of the same transmission chain remained unobserved.^18^ For this reason, population-level inferences into the drivers of pathogen transmission focus on analyses that seek to harness the information contained in phylogenetic trees spanning all available samples,^19–22^ particular parts of phylogenetic trees,^23,24^ or a larger number of phylogenetically reconstructed transmission pairs.^17,25,26^ The latter approaches have proven particularly useful when additional data provide insights into the direction of transmission,^10,27^ as then flexible and computationally efficient regression methods using attributes of the likely source and recipient can be used to quantify transmission flows.^28,29^ Large sets of phylogenetic transmission pairs are typically identified using genetic distances between pathogen sequences or patristic distances along lineages in phylogenetic trees, sometimes coupled with additional criteria including the statistical support that the two individuals are part of the same sub-tree of the true, unknown phylogeny, or the depth of the lineage separating the two individuals, often expressed in units of calendar time.^30^ In practice, these linkage criteria are often loosely justified, but especially those based on evolutionary distances can be more firmly grounded in statistical models on the expected number of genetic mutations under a generative evolutionary clock model.^31,32^ Clock models describe the genetic distance between two sequences in terms of the amount of time elapsed since the lineages leading to the two observed sequences diverged and are used widely to characterize pathogen evolution, including, for example, the evolution of novel severe acute respiratory syndrome coronavirus 2 (SARS-CoV-2) variants in immuno-compromised patients.^33^ The challenge is to define the time elapsed, since the divergence time of the lineages leading to the observed sequences is unknown. We address this, using HIV as an example, by leveraging additional data that can be used to estimate the time since infection and approximate the time elapsed as the interval between the infection event and the sampling time of two observed sequences, which assumes that the two lineages diverged at the infection event (Figure 1). We then express the likelihood that observed genetic distances evolved within a specified time elapsed between two individuals under a standard evolutionary clock model. This approach can account for substantial natural heterogeneity in time elapsed when attempting to interpret genetic distances arising from variation in when HIV is transmitted, how late individuals are diagnosed, and when diagnosed individuals’ samples are sequenced. We then embed this transmission pair likelihood into a two-component Bayesian mixture model (BMM) to estimate posterior probabilities that pairs of individuals are actual transmission pairs, relative to a background noise distribution on genetic distances and time elapsed. We will see that the posterior transmission pair probabilities are themselves uncertain and so are not of immediate interest, but by aggregating the posterior transmission pair probabilities we can identify and quantify the drivers of transmission at population-level.

BMMs are widely used to classify points into latent components of similar data,^34^ characterized by a distinct probability distribution. As such, BMMs are a natural starting point for interpreting two-dimensional point patterns of genetic distances and time elapsed. We limit ourselves to allocating points probabilistically into two components—a signal and a background component—rather than exploring high or infinite-dimensional components,^35^ as our primary interest centers on the population-level drivers of infection and not the point classifications. More fundamentally, unrelated pairs of individuals can have genetic distances and times elapsed that are compatible with the HIV evolutionary rate. This is a challenge because the number of all possible pairwise combinations of individuals far exceeds the number of transmission pairs. We adopt previous work that attaches generalized linear predictors to the BMM mixing weights to improve classification accuracy^36^ and other big data reduction techniques, circumventing highly imbalanced classification problems. Sections 2.1 to 2.3 lay out the notation that we use to describe unknown transmission events and transmission flows, and the data available to estimate these. Section 2.4 introduces the Bayesian hierarchical evolutionary clock model that we use to represent the relationship between viral HIV sequences and the time elapsed between them. In Sections 2.5 to 2.7, we integrate the evolutionary clock model as a signal component of increasingly complex BMMs and describe how we estimate population-level transmission flows and population-level sources of transmission. Section 3 assesses the performance of the mixture model on simulated data, characterizes the accuracy of phylogenetic source attribution with BMMs, and presents techniques to improve classification accuracy when the number of unrelated pairs far exceeds the number of actual transmission pairs. In Section 3.5, we apply the method to characterize the age-specific drivers of HIV transmission between 2010 and 2021 among men who have sex with men (MSM) in Amsterdam, the Netherlands, who were part of phylogenetically reconstructed transmission chains. Finally, Section 4 summarizes our findings and discusses the application of our method to other pathogens.

## 2. Methods

### 2.1. Target quantities

Consider a population of n individuals infected with a pathogen. Of these, m new cases were acquired in a specific time period T , where m≤n . The estimated infection date of an individual i is denoted by $T_{i}$ . We are interested in characterizing the transmission events to the m new cases in T , and denote these with $Z_{ij}=1$ if i infected j for i=1,…,n and j=1,…,m , and $Z_{ij}=0$ otherwise. We refer to i as a source or a transmitting partner of j when $Z_{ij}=1$ , and to i and j as linked when either $Z_{ij}=1$ or $Z_{ji}=1$ , and to i and j as unlinked if both $Z_{ij}=0$ and $Z_{ji}=0$ . The unknown n×m matrix Z is commonly referred to as the adjacency matrix. We primarily wish to characterize population-level transmission flows between groups of individuals specified by distinct demographic, behavioral, or clinical covariates. We denote a partition of the study population with A , and population groups in this partition by a,b∈A , and aggregate over individuals in any of these population groups, which we denote by i,j∈a . We quantify the population-level transmission counts in time period T between population strata with $Z_{ab}=\sum_{i\in a,j\in b}Z_{ij}$ for all a,b∈A . We are primarily interested in the relative contributions of population groups to the transmission counts, and our target quantities are the population-level transmission flows from group a to group b , the transmission sources from group a among infections in group b , and the transmission sources from group a in the entire population, respectively, defined by

(1a) $$\begin{array}{rl} \pi_{ab} & =Z_{ab}/(\sum_{c,d\in A}Z_{cd}) \end{array}$$

(1b) $$\begin{array}{rl} \delta_{ab} & =Z_{ab}/(\sum_{c\in A}Z_{cb}) \end{array}$$

(1c) $$\begin{array}{rl} \delta_{a} & =(\sum_{b\in A}Z_{ab})/(\sum_{c,d\in A}Z_{cd}) \end{array}$$

### 2.2. Observations

A subset of infected individuals are diagnosed, and we denote the number of diagnosed individuals infected with the pathogen by $n^{D}$ and the number of diagnosed new cases in time period T by $m^{D}$ . In the Netherlands, all individuals diagnosed with HIV enter the open longitudinal ATHENA cohort except a small proportion of patients who opt out.^37^ Using available clinical and demographic biomarker data, the time of infection of diagnosed individuals can be estimated,^38,39^ which we denote by ${\hat{T}}_{i}$ for $i=1,\ldots ,n^{D}$ . Then, we consider as potential sources of transmission to a new case all diagnosed individuals with an infection date preceding the case. We denote a “potential source” by

(2) $$Y_{ij}^{D}=\left\{ \begin{array}{l} 1\text{ if }{\hat{T}}_{i}<{\hat{T}}_{j}\\ 0\text{ if }{\hat{T}}_{i}\geq {\hat{T}}_{j} \end{array}\right.$$

where $i=1,\ldots ,n^{D}$ and $j=1,\ldots ,m^{D}$ .

All potential sources are epidemiologically possible sources of infection, based on the times elapsed and the available biomarker data used to estimate the times elapsed. We use the term “potential sources” to describe epidemiologically possible sources, which we distinguish from “phylogenetically possible” sources, and “likely sources” following inference under the Bayesian model that we describe further below.

The values of the $n^{D}\times m^{D}$ matrix $Y^{D}$ are observed. Often, diagnosis times are used in lieu of infection time estimates;^25,40^ in many cases, these data are already highly informative for estimating Z , for example, in the case of new influenza outbreaks in school settings.^41^ In most settings, however, such as the spread of HIV at the city level or nationally,^17,25,39^ the number of potential sources to any newly diagnosed case is typically very large and thus not very informative on Z .

It is often possible to narrow down the potential sources of new cases based on demographic and clinical data, for example:

- exclude pairs with a time elapsed >16 years, since each individual is likely to show symptoms within 8 years of seroconversion;^42^
- using mortality data, exclude pairs in which the potential source died before the estimated infection date of the recipient;^17^
- using migration and mobility data, exclude pairs in which the potential source did not reside in the study population by the estimated infection date of the recipient;^43,44^ and
- using clinical data, exclude pairs with evidence that the potential source was not infectious on the infection date of the recipient. In our HIV case study, we estimated subject-specific viral load curves with LOESS smoothers to longitudinally collected viral load measurements and considered individuals with viral loads below 200 copies of virus per milliliter blood as non-infectious.^45,46^

However, in our case study, the number of potential sources for each new case continues to be very large with these exclusion criteria applied, which motivates us to consider pathogen sequence data.

A smaller subset of infected individuals also have a pathogen sequence sampled, and we denote the number of diagnosed individuals with a sampled pathogen by $n^{S}$ and the number of diagnosed new cases with a sampled pathogen in time period T by $m^{S}$ . In the Netherlands, all newly diagnosed patients should have a partial HIV *polymerase* (*pol*) sequence sampled to determine potential drug resistance and suitable combination antiretroviral treatment for therapy.^47^ Using population-level pathogen data, we construct HIV phylogenetic trees and perform ancestral state reconstruction with additional background sequences from outside of the study population to identify phylogenetically likely transmission chains circulating in the study population.^39^ We reconstructed maximum-likelihood phylogenies with *FastTree*,^48^ which runs highly efficiently for a large number of taxa and reconstructed ancestral states with *phyloscanner*,^10^ but other approaches could also be used.^49–53^ Given the large molecular genetic diversity of HIV, these steps are performed separately for each of the predominant HIV subtypes and circulating recombinant forms (CRFs) in the population.^39,54^ Phylogenetically likely transmission chains are defined as groups of connected tips and internal nodes with corresponding ancestral states. In the case of HIV, most phylogenetically observed transmission chains are typically of size one, with no evidence of onward transmission in the study population. We focus only on those phylogenetically likely transmission chains with at least two members, defined by $C=(C_{1},\ldots ,C_{n^{C}})$ , each containing a vector of vertices corresponding to its members. We denote a “phylogenetically possible” source of a new case by

(3) $$Y_{ij}^{S}=\left\{ \begin{array}{l} 1\text{ if }{\hat{T}}_{i}<{\hat{T}}_{j}\cap \{i,j\}\in C_{g}\text{ for any }g=1,\ldots ,n^{C}\\ 0\text{ otherwise} \end{array}\right.$$

where $i=1,\ldots ,n^{S}$ and $j=1,\ldots ,m^{S}$ . The values of the $n^{S}\times m^{S}$ matrix $Y^{S}$ are observed. For ease of reference we denote the phylogenetically possible transmission pairs by $P=\{i=1,\ldots ,n^{S},j=1,\ldots ,m^{S}|Y_{ij}^{S}=1\}$ , the phylogenetically possible sources of j by $P_{j}=\{i=1,\ldots ,n^{S}|Y_{ij}^{S}=1\}$ , and the total number of phylogenetically possible transmission pairs by $n^{P}$ .

### 2.3. Accounting for time elapsed when interpreting the patristic distance of virus from two individuals

It is common to base phylogenetic inference of linkage on the number $D_{ij}$ of nucleotide mutations between pathogen sequences of i and j that occur along the shortest path in an estimated pathogen phylogeny. The patristic distance is non-negative and can be calculated with the R package adephylo^55^ for all $i,j=1,\ldots ,n^{S}$ , but here we restrict attention to $i\in P_{j}$ and $j=1,\ldots ,m^{S}$ . To account for differences in times from infection to sampling, we argue in this paper that inferences should also account for the time elapsed between pathogen samples since their divergence. To this end, we denote the sequence sampling dates of a sampled individual i by $S_{i}$ , where $S_{i}>{\hat{T}}_{i}$ . To calculate the time elapsed, we make the approximation that the pathogen lineages leading to the observed samples in i and j diverged at the transmission event. Then, in the case that the sampling date of the source was before the estimated infection date of the recipient, we define time elapsed, $T_{ij}^{e}$ by

(4) $$T_{ij}^{e}=({\hat{T}}_{j}-S_{i})+(S_{j}-{\hat{T}}_{j})$$

In case the sampling date of the source was after the estimated infection date of the recipient, as illustrated in Figure 1, we have

(5) $$T_{ij}^{e}=(S_{i}-{\hat{T}}_{j})+(S_{j}-{\hat{T}}_{j})$$

So, both cases can be subsumed under

(6) $$T_{ij}^{e}=|S_{i}-{\hat{T}}_{j}|+(S_{j}-{\hat{T}}_{j})$$

for all $i,j=1,\ldots ,n^{S}$ , but again we will restrict attention to $i\in P_{j}$ and $j=1,\ldots ,m^{S}$ . Pathogens mutate over time, and so we expect that including the time elapsed into inferences will improve the estimation of Z , especially for rapidly evolving pathogens such as HIV.^56^ The data for inferring transmission flows in the study population are the patristic distances and estimated times elapsed across phylogenetically possible sources of new cases,

(7) $$X=\{D_{ij},T_{ij}^{e}\;|\;(i,j)\in P\}$$

### 2.4. Signal component for the mixture model

We next develop a likelihood model for patristic distances and times elapsed from data of known transmission pairs, which will serve as the signal component of the BMM. A detailed knowledge of transmission chains is rare. However, one clinical investigation^57^ in Belgium previously led to the characterization of the direction of transmission between individuals of one HIV transmission chain and the timing of transmission events. Subsequently, a large number of viral sequences were obtained for in-depth molecular analysis of this chain,^58^ and we developed the signal component of the BMM based on these data within a Bayesian random effects modeling framework due to the extensive variation in within-host viral evolution in these data.^59^ In the Belgian study, HIV *pol* sequences were sampled for each individual in the known transmission chain from multiple time points. We therefore define each sequence pair for a source i and recipient j by $k=1,\ldots ,K_{ij},$ where $K_{ij}$ is the total number of sequence pairs available for each transmission pair ij . We also denote the entire Belgian transmission chain data with B . Our resulting Bayesian hierarchical evolutionary clock model is

(8a) $$\begin{array}{rl} D_{ijk} & ∼\text{Gamma}(\alpha_{ijk},\beta_{ij}) \end{array}$$

(8b) $$\begin{array}{rl} \alpha_{ijk} & =\mu_{ijk}\beta_{ij} \end{array}$$

(8c) $$\begin{array}{rl} \mu_{ijk} & =(\gamma +\gamma_{ij})T_{ijk}^{e} \end{array}$$

(8d) $$\begin{array}{rl} \beta_{ij}^{-1} & =\phi +\phi_{ij} \end{array}$$

where $D_{ijk}$ is the kth patristic distance and $T_{ijk}^{e}$ is the kth time elapsed for pair ij . The Gamma observation likelihood is in shape-scale parameterization such that its mean equals $\mu_{ijk}=\alpha_{ijk}/\beta_{ij}$ and its variance equals $\alpha_{ijk}/\beta_{ij}^{2}$ , and the prior densities are

(9a) $$\begin{array}{rl} \log\;\gamma & ∼N(\log\;(10^{-2.5}),0.2^{2}) \end{array}$$

(9b) $$\begin{array}{rl} \log\;\gamma_{ij} & ∼N(0,\sigma_{\gamma }^{2}) \end{array}$$

(9c) $$\begin{array}{rl} \sigma_{\gamma } & ∼\text{Exp}(10) \end{array}$$

(9d) $$\begin{array}{rl} \log\;\phi & ∼N(0,5^{2}) \end{array}$$

(9e) $$\begin{array}{rl} \log\;\phi_{ij} & ∼N(0,\sigma_{\phi }^{2}) \end{array}$$

(9f) $$\begin{array}{rl} \sigma_{\phi } & ∼\text{Exp}(10) \end{array}$$

Here, $\mu_{ijk}$ denotes the expected evolutionary distance in the kth sequence pair for source i and recipient j and given the time elapsed $T_{ijk}^{e}$ , where γ corresponds to the overall log mean evolutionary rate across pairs and is given an informative prior,^60^ and $\gamma_{ij}$ are zero-mean random effects specific to transmission pair ij . The parameter ϕ corresponds to the mean degree of dispersion across pairs, while $\phi_{ij}$ are pair-specific zero-mean random effects. The model for the signal component may be adapted for other applications, for example, if there is a consensus of evidence supporting non-linear evolutionary rates over time (see Supplemental Material S2).

**Figure 2.**
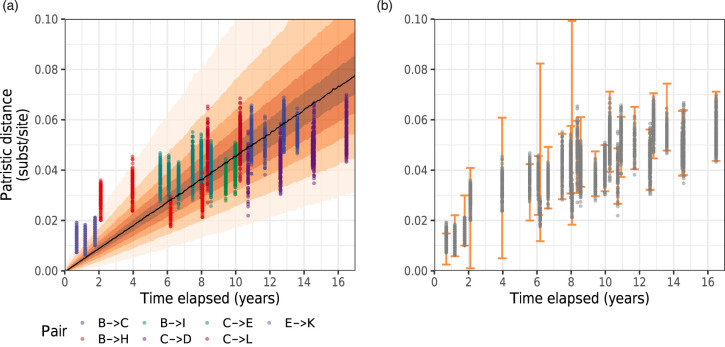
Belgian transmission chain data and model fit of evolutionary clock model. (a) Patristic distances between pairs of sequences from epidemiologically confirmed transmission pairs in Belgium, against time elapsed. Colors denote transmission pairs. Orange ribbons correspond to quantiles (at 10% increments between 10% and 90%, in addition to 95% credible intervals) of the posterior predictive distribution of the average evolutionary rate across pairs in the hierarchical clock model. (b) Posterior predictive 95% credible intervals of patristic distances by time elapsed (orange) for known transmission pairs, with data points overlaid. Transmission pair B → H leads to large uncertainty intervals at times elapsed 2–8 but are retained in the model, which allows for between-pair heterogeneity, to increase the number of training data points.

The model in (8) and (9) was fitted with cmdstanr v.2.28.1 with four chains of 2000 samples each, including a burn-in of 500, and fitted the data well, as shown by Figure 2.

The predictive distribution of the shape and scale parameters of the model at a given time elapsed for a new pair, not in the training dataset, $T_{ij}^{e}$ , is given by

(10) $$\begin{array}{rl} f(\alpha_{ij}^{*}(T_{ij}^{e}),\beta_{ij}^{*}|B) & =\int p(\alpha_{ij}^{*}(T_{ij}^{e}),\beta_{ij}^{*}|\gamma_{ij}^{*},\phi_{ij}^{*})\;p(\gamma_{ij}^{*},\phi_{ij}^{*}|\gamma ,\phi ,\sigma_{\gamma },\sigma_{\phi })\\ & \;\cdot p(\gamma ,\phi ,\sigma_{\gamma },\sigma_{\phi }|B)\;d(\gamma_{ij}^{*},\phi_{ij}^{*}) \end{array}$$

We found that the predictive distribution obtained by replacing the posterior distributions for the hyper-parameters with their posterior medians was almost identical to (10) (Supplemental Figure S1), and for computational simplicity we used the latter.

### 2.5. Two-component mixture model to identify transmission flows

We next incorporate the fitted model described by (8) and (9) as a signal component for phylogenetically likely transmission pairs into a two-component BMM. For ease of read, we will from now on suppress in the notation that the signal component was derived on the data B . We emphasize as others^61^ that it is not possible to prove transmission between two individuals from the phylogenetic data, and we will throughout use the BMM transmission status allocations to each observation only as latent variables from which we deduce population-level patterns of transmission dynamics. The mixture model is constructed as follows. Conditional on the (unknown) transmission event having occurred ( $Z_{ij}=1$ ), we model the observed patristic distances for (i,j)∈P through

(11) $$\begin{array}{rl} & p(D_{ij}|T_{ij}^{e},Z_{ij}=1)=\int \text{Gamma}(D_{ij}|\alpha^{*}(T_{ij}^{e}),\beta^{*})\;f(\alpha^{*}(T_{ij}^{e}),\beta^{*})\;d(\alpha^{*}(T_{ij}^{e}),\beta^{*}) \end{array}$$

where $f(\alpha^{*}(T_{ij}^{e}),\beta^{*})$ is the posterior predictive distribution (10) at the estimated time elapsed $T_{ij}^{e}$ . If there was no transmission event between individuals i and j , we model

(12) $$p(D_{ij}|T_{ij}^{e},Z_{ij}=0)=\text{Uniform}(0,d^{\max})$$

where $d^{\max}$ is a suitably chosen large value, here $d^{\max}=0.2$ . We assume that the background distribution does not depend on time elapsed, but other choices are possible.

The resulting BMM is

(13a) $$\begin{array}{rl} p(D_{ij}|T_{ij}^{e}) & =\omega \;p(D_{ij}|T_{ij}^{e},Z_{ij}=1)+(1-\omega )\;p(D_{ij}|Z_{ij}=0) \end{array}$$

(13b) $$\begin{array}{rl} \text{logit}(\omega ) & ∼N(0,2^{2}) \end{array}$$

for all (i,j)∈P . Model (13) integrates out the latent transmission status variables Z and implicitly assumes that $Z_{ij}∼\text{Bernoulli}(\omega )$ , where ω is the mixing weight.^34^ Since ω is assumed to be the same across all possible transmission pairs, we refer to (13) as the “vanilla mixture model,” indicating it has no additional data informing ω . Since ω is likely to decrease as the number of pairs under consideration increases, the variance for the prior on ω should be chosen to ensure support over the smallest value it could plausibly take, calculated as the number of incident cases divided by the total possible pairs without any exclusion criteria, N/N(N−1) . Note that the hyper-parameters of the signal component are specified through the data from the known Belgian transmission chain and the background component has no parameters, so the only parameters to be inferred are the mixing weight and the signal component random effects.

### 2.6. Estimating transmission flows from the latent, likely transmission pairs

The primary purpose of the fitted BMM is to characterize population-level transmission flows between groups of individuals specified by distinct demographic, behavioral, or clinical covariates. We achieve this by considering all pairwise combinations of sampled individuals with source and recipient attributes a and b according to their posterior probabilities of being a transmission pair. There are two cases to consider and we begin by assuming that each sampled recipient j has exactly one sampled possible source case i . Following notation in (1), the posterior transmission flows from group a to group b under the mixture model are

(14a) $$\begin{array}{rl} Z_{ab}|X & =\sum_{i\in a}\sum_{j\in b}\rho_{ij}|X \end{array}$$

(14b) $$\begin{array}{rl} \rho_{ij}|X & =\frac{\omega \;p(D_{ij}|T_{ij}^{e},Z_{ij}=1)}{\omega \;p(D_{ij}|T_{ij}^{e},Z_{ij}=1)+(1-\omega )\;p(D_{ij}|Z_{ij}=0)} \end{array}$$

(14c) $$\begin{array}{rl} \omega & ∼p(\omega |X) \end{array}$$

for all a,b∈A , where $\rho_{ij}$ denotes the posterior probability that pair ij is a transmission pair, also called the component membership probability in more general applications, and p(ω|X) denotes the posterior distribution of the mixing weights. If a pair involving the only phylogenetically possible source and the incident individual is outside the signal component, then it is likely incompatible with being a true transmission pair. Therefore, even if there is only one phylogenetically possible source, it is possible that the proposed approach infers from the data that they are unlikely to be the true transmission source.

For recipient j , if the data contain more than one phylogenetically possible source $i_{1},\ldots ,i_{L_{j}}$ with $n_{j}^{P}>1$ total possible sources, then the posterior transmission probabilities (14b) are obtained by considering that transmission might have occurred from exactly one or none of the $n_{j}^{P}$ phylogenetically possible sources. For this, we quantify the posterior probability of events of the form $\{Z_{\left(i_{1},j\right)}=0\cap \ldots \cap Z_{\left(i_{u-1},j\right)}=0\cap Z_{\left(i_{u},j\right)}=1\cap Z_{\left(i_{u+1},j\right)}=0\cap \ldots \cap Z_{\left(i_{n_{j}^{P}},j\right)}=0$ for $u=1,\ldots ,n_{j}^{P}$ , obtaining

(15) $$\begin{array}{rl} & \rho_{i_{u},j}|X\\ & \;=(\omega \;p(D_{i_{u}j}|T_{i_{u}j}^{e},Z_{i_{u}j}=1)\prod_{v\neq u}(1-\omega )\;p(D_{i_{v}j}|T_{i_{v}j}^{e},Z_{i_{v}j}=0))/\\ & \;[\sum_{w=1}^{n_{j}^{P}}(\omega \;p(D_{i_{w}j}|T_{i_{w}j}^{e},Z_{i_{w}j}=1)\prod_{v\neq w}(1-\omega )\;p(D_{i_{v}j}|T_{i_{v}j}^{e},Z_{i_{v}j}=0))\\ & \;\;+\prod_{v=1}^{n_{j}^{P}}(1-\omega )\;p(D_{i_{v}j}|T_{i_{v}j}^{e},Z_{i_{v}j}=0)] \end{array}$$

for all $u=1,\ldots ,n_{j}^{P}$ and then evaluate $Z_{ab}|X=\sum_{i_{u}\in a}\sum_{j\in b}\sum_{u=1}^{n_{j}^{P}}\rho_{i_{u}j}|X$ .

### 2.7. Incorporating additional covariates

We next considered incorporating additional individual-level covariates as predictors of the BMM mixing weights to better separate true transmission pairs from those with false transmission pair signal, that is, truly unlinked pairs of individuals that fall into the signal component of the mixture model (11). We denote p -dimensional covariates for all $n^{P}$ phylogenetically possible pairs with $C\in R^{n^{P}\times p}$ . These could be the age of each individual in a pair, socio-demographic characteristics, place of birth, or even pairwise properties such as being part of the same household. This updates the vanilla mixture model (13) to the “covariate” mixture model

(16a) $$\begin{array}{rl} p(D_{ij}|T_{ij}^{e}) & =\omega_{ij}\;p(D_{ij}|T_{ij}^{e},Z_{ij}=1)+(1-\omega_{ij})\;p(D_{ij}|Z_{ij}=0) \end{array}$$

(16b) $$\begin{array}{rl} \text{logit}(\omega_{ij}) & =\eta_{0}+C_{ij}\eta \end{array}$$

(16c) $$\begin{array}{rl} \eta_{0} & ∼N(0,2^{2}) \end{array}$$

(16d) $$\begin{array}{rl} \eta & ∼N(0,1) \end{array}$$

where $C_{ij}$ is the row of the matrix of covariates corresponding to the ijth pair and $\eta_{0}\in R$ and $\eta \in R^{p}$ are additional model parameters.

In many cases, the covariates can be continuous, such as the age of the likely source and the likely recipient individual in the ijth pair. In these settings, we model the dependency of the mixing weights on the covariates through univariate or bivariate random functions. In the latter case, for computational efficiency, we use zero-mean two-dimensional Hilbert-space Gaussian process (HSGP) approximations, which scale more efficiently with a large number of observations compared to bivariate Gaussian process priors.^29,62,63^ This updates the vanilla mixture model to the “HSGP random function” mixture model

(17a) $$\begin{array}{rl} p(D_{ij}|T_{ij}^{e}) & =\omega_{ij}\;p(D_{ij}|T_{ij}^{e},Z_{ij}=1)+(1-\omega_{ij})\;p(D_{ij}|Z_{ij}=0) \end{array}$$

(17b) $$\begin{array}{rl} \text{logit}(\omega_{ij}) & =\eta_{0}+f(x_{ij,1},x_{ij,2}) \end{array}$$

(17c) $$\begin{array}{rl} f & ∼\text{HSGP}(0,k((x_{1},x_{2}),(x_{1},x_{2}{)}^{\prime })) \end{array}$$

(17d) $$\begin{array}{rl} k((x_{1},x_{2}),(x_{1},x_{2}{)}^{\prime }) & =\alpha \exp\;\left(\frac{(x_{1}-x_{1}^{\prime }{)}^{2}}{2\ell_{x_{1}}^{2}}+\frac{(x_{2}-x_{2}^{\prime }{)}^{2}}{2\ell_{x_{2}}^{2}}\right) \end{array}$$

(17e) $$\begin{array}{rl} \eta_{0} & ∼N(0,2^{2}) \end{array}$$

(17f) $$\begin{array}{rl} \alpha & ∼N(0,0.15^{2}) \end{array}$$

(17g) $$\begin{array}{rl} \ell_{x_{1}},\ell_{x_{2}} & ∼\text{Inv-Gamma}(5,5) \end{array}$$

where α∈R and $\ell_{x_{1}},\ell_{x_{2}}\in R^{+}$ are additional model parameters. Further details on the HSGP regularizing prior density are in the Supplemental Material S3. Transmission flows are calculated from the posterior as before (15).

### 2.8. Numerical inference

Throughout, the model was fitted with the Stan probabilistic computing language using the cmdstanr interface, v2.28.1, with four chains of 2000 samples each, and a burn-in of 500. Code is available at github.com/ MLGlobalHealth/source.attr.with.infection.time . There were no observed divergences, and the minimum number of effective samples across all parameters was 2065 on the simulated data and 3492 on our application to Amsterdam data; see also the Supplemental Material S1 for diagnostic plots.

### 2.9. Simulations to evaluate estimation performance

We assessed the performance of the mixture models for estimating transmission flows and transmission sources on simulated HIV transmission networks derived from the discrete-time individual-based HIV epidemic model (PopART-IBM), which was developed in the context of the HPTN071/PopART HIV combination intervention prevention trial.^64^ The model is informed by data collected from surveys, health care facilities, and community healthcare workers delivering interventions as part of the HPTN071 trial. The model parameters were calibrated to age-sex-specific data on incidence, prevalence, antiretroviral therapy (ART) uptake, and viral suppression from a representative cohort at the level of communities participating in the trial. Simulated transmission events depend on individual-level parameters including age, sex, set point viral load, and CD4 counts of the transmitter (Supplemental Table S1). The simulation begins with the starting population size in 1900, and the epidemic is seeded randomly with infectious cases in 1965–1970. The model runs until 2020 and returns a large number of separate, simulated transmission chains. For each new case, sequence sampling dates were simulated using a Weibull distribution. For each transmission pair, the time elapsed was calculated and patristic distances were simulated from the signal distribution (8). For unlinked pairs, patristic distances were simulated from the uniform background distribution (12). Full details are presented in Supplemental Material S4.

### 2.10. Evaluating estimation performance

For each of the models (13), (16), and (17), the accuracy of the target quantities (1) from the posterior was evaluated by comparing the mean absolute error (MAE), defined for each Monte Carlo sample, to the corresponding true quantity in the simulated data among known transmission pairs. For (1a), $\text{MAE}=\sum_{a,b\in A}|\pi_{ab}-\pi_{ab}^{*}|/|A|$ , where $\pi_{a,b}^{*}$ are the true simulated flows from group a to group b . The posterior MAE was summarized by taking the median and 95% quantiles. The MAE for the remaining target quantities was evaluated analogously.

## 3. Results

### 3.1. Constructing the signal component of the BMM

We first fitted the Bayesian hierarchical evolutionary clock model to patristic distances and time elapsed from 5186 sequences from 10 individuals in a well-characterized HIV transmission chain from Belgium.^57,58^ Maximum-likelihood viral phylogenies were reconstructed with RAxML v7.4.2.^65^ Excluding pairs involving one multi-drug-resistant individual, patristic distances between 2807 sequence combinations from eight individuals involved in seven known transmission events were computed from the reconstructed phylogeny. Infection times were ascertained to narrow time ranges of up to a few months in the study, and we used the midpoint of these uncertainty ranges. The time elapsed was calculated according to (6) for each combination of viral sequences from the recipient and transmitting partner of each transmission event. Figure 2(a) illustrates the patristic distances relative to the time elapsed for all 2807 pairwise sequence combinations, with data corresponding to each of the seven transmission events shown in a different color. We found substantial heterogeneity in the rate of evolution across transmission pairs, prompting us to use the Bayesian hierarchical model (8) and (9) to estimate an overall mean evolutionary clock and associated uncertainty range. The model fit the data well, with 96% of the observed genetic distances within 95% posterior predictive credible intervals (CrIs; Figure 2(b)). The estimated overall mean evolutionary rate was $4.5\times 10^{-3}$ [95% CrI $3.5\times 10^{-3}$ – $5.6\times 10^{-3}$ ] substitutions per site per year, compatible with previously published estimates.^58^ Figure 2(a) shows in orange the shape of the estimated posterior predictive distribution of patristic distances under the model for each 0.1-year increment in time elapsed, which defines the signal component of the BMM for phylogenetic source attribution. For reasons of parsimony, we chose a uniform distribution as the background component (see Section 2).

### 3.2. False transmission pair signal in the BMM signal component

**Figure 3.**
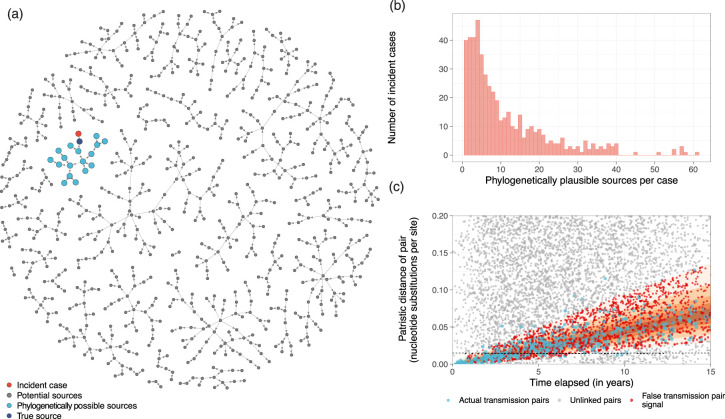
Phylogenetically possible sources and false transmission pair signal in an HIV epidemic simulation. (a) Sample of transmission chains containing one of the 500 most recent incident cases in the simulation generated under the PopART-IBM. One of the recent cases is highlighted in red, a random sample of their potential sources in gray, which correspond to sampled participants who are part of a distinct phylogenetically observed transmission chain, all phylogenetically possible sources in light blue, and the actual transmitting partner of the simulation in dark blue. (b) Number of all phylogenetically possible sources for the 500 most recent incident cases in the simulation. (c) Patristic distances and time elapsed between phylogenetically possible sources and each of the 500 most recent incident cases in the simulation. Actual transmission pairs in the simulation are shown in blue. Pairs that exhibited false transmission pair signal are shown in red. These are defined as phylogenetically possible unlinked pairs with patristic distances that fall within the 95% quantiles of the posterior predictive distribution of the distances for a given time elapsed from the fitted molecular clock model. All other unlinked pairs are in gray.

We next sought to assess the estimation accuracy of the two-component vanilla BMM (13) on simulated data generated under an individual-based HIV epidemic model developed to represent contemporary African HIV epidemics and used to interpret the outcomes of the HIV prevention trial network trial 071 (HPTN071/PopART).^64^ The model simulated transmission dynamics over 55 years from 1965 in a starting population of 32,217, initializing the epidemic with 148 transmission events to randomly chosen individuals in the first five years under default input parameters (see Supplemental Material S4). The model simulated 34,961 transmission events. For ease of tractability, we considered only the most recent 500 incident cases between 9 June 2017 and 31 December 2019, which were distributed over 276 distinct transmission chains (Figure 3(a)). We assumed that a pathogen sequence was sampled for all individuals with HIV. Since 2005, there have been 9355 potential sources with infection dates that preceded those of a new case between mid-2017 and the end of 2019. These formed 4,552,006 unique pairwise combinations of potential heterosexual transmission pairs with the 500 incident cases. In total, 768,257 potential pairs were excluded based on very large time elapsed, including 39 actual transmission events, due to the epidemic model simulating individual infection times which can lead to unrealistic values from the tails of the distribution in some cases (see Section 2). The 461 actual transmission events thus comprised 0.012% among all potential transmission pairs, rendering the unlabeled classification task of identifying true transmission pairs highly imbalanced.

Individuals were grouped into distinct phylogenetic transmission networks, reducing the number of heterosexual, phylogenetically possible transmission pairs to 5519, with the 461 actual transmission events comprising 8.3% of the remaining pairs, a 600-fold improvement in balance. Each newly acquired case had on average 9,104 potential sources and 11.2 phylogenetically possible sources after excluding potential sources not within the same transmission chain (Figure 3(b)). Figure 3(c) illustrates how the quantitative data on patristic distances and time elapsed narrow down further the likely transmitters among pairs that fall into the signal component of the BMM. Importantly, a considerable fraction of the transmission events in the simulation (shown in red in Figure 3(c)), and their true sources, were associated with late diagnosis, which resulted in a large time elapsed. Application of standard phylogenetic selection criteria for reconstructing transmission pairs, such as a maximum patristic distance of 1.5% (i.e. 1.5 substitutions per 100 nucleotides,^66–69^ indicated by the horizontal line in Figure 3(c)), would have excluded these pairs, introducing selection bias. By accounting for time elapsed in the phylogenetic source attribution problem, we avoid this selection bias. However, the inference problem remains challenging, since 37% of phylogenetically possible pairs exhibited false transmission pair signal, defined as truly unlinked pairs that fall into the 95% quantiles of patristic distances for a given time elapsed under the BMM signal component.

### 3.3. Accuracy in phylogenetic source attribution with the BMM with little false transmission pair signal

**Figure 4.**
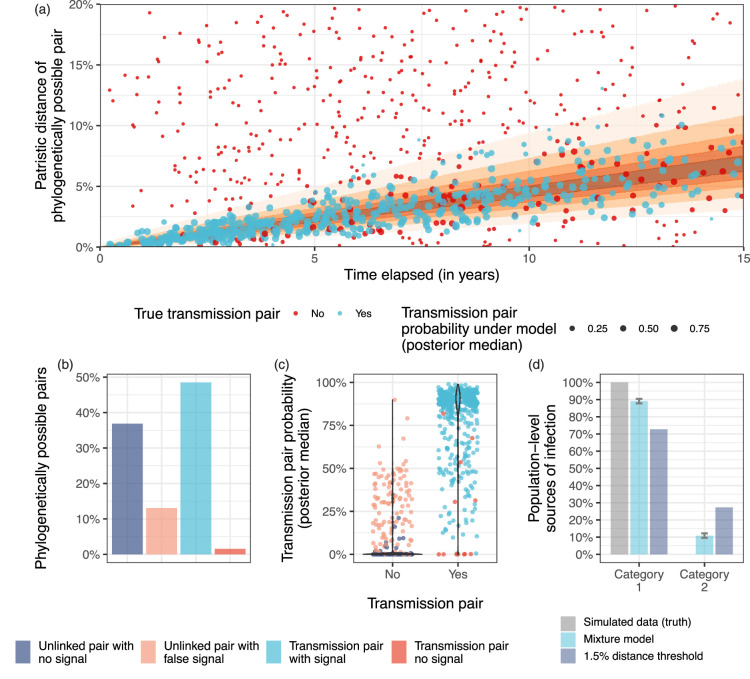
Source attribution with the vanilla Bayesian mixture model (BMM) in the case of two competing phylogenetically possible sources per new case. From the HIV epidemic simulation, only two competing phylogenetically possible sources for each of the 500 most recent cases were retained to assess source attribution in an idealized scenario. (a) Patristic distances and time elapsed for each phylogenetically possible pair, with true transmission pairs shown in blue and unlinked pairs in red. The size of observations corresponds to their posterior probability of being a true transmission pair inferred from the vanilla BMM. (b) Empirical proportion of pairs with and without transmission pair signal (based on 95% quantiles of a molecular clock), for actual transmission pairs and unlinked pairs among the phylogenetically possible pairs. (c) Posterior probability of being a transmission pair, by actual transmission pairs and unlinked pairs. (d) Posterior median estimates of transmission flows and 95% credible intervals under the vanilla BMM, compared to using a 1.5% threshold on patristic distances, and simulated ground truth.

With these statistical challenges in mind, we first evaluated the estimation accuracy of the BMM in the idealized scenario that the newly acquired cases had each on average two phylogenetically possible sources, including the true transmitting partner. The unlinked pairs were sampled randomly to achieve the desired balance. On these pairs, we considered the following binary source attribution problem. In each of the 461 transmission events, the true transmitting partners were allocated to have the same population-level characteristic “category 1,” while all other phylogenetically possible but unlinked sources were allocated to “category 2.” In this simulation, all phylogenetically possible pairs had a small patristic distance ( <0.2 ) (Figure 4). Of these, 13% of pairs were unlinked with false transmission pair signal (i.e. lying within the 95% quantiles of the BMM signal component), and 1.5% were true transmission pairs with no transmission pair signal (i.e. lying outside the 95% quantiles of the signal component) (Figures 4(b)). We then fitted the vanilla BMM (13) to these data, aiming to estimate that 100% of transmissions originated from category 1. Figure 4(c) illustrates the posterior probabilities that each pair is classified by the BMM as a true transmission pair. A small number of true transmission pairs exhibiting no signal were inferred by the model to be likely transmission pairs (posterior transmission pair probability >0.5 ), likely the result of lying close to the edges of the posterior predictive distribution of the signal component. Weighting each phylogenetically possible pair by the posterior transmission pair probabilities, an estimated 89% (88%–90%) of transmissions originated from category 1 (Figure 4(d)), implying an MAE for the sources estimated among the entire population (1c) of 11% [10%–12%]). For comparison, if pairs were classified as phylogenetically likely transmission pairs as commonly done based on patristic distances <1.5%,^66–69^ 73% of transmissions were inferred to originate from category 1, implying an MAE of 27%.

### 3.4. Accuracy in phylogenetic source attribution with the BMM with substantial false transmission pair signal

**Figure 5.**
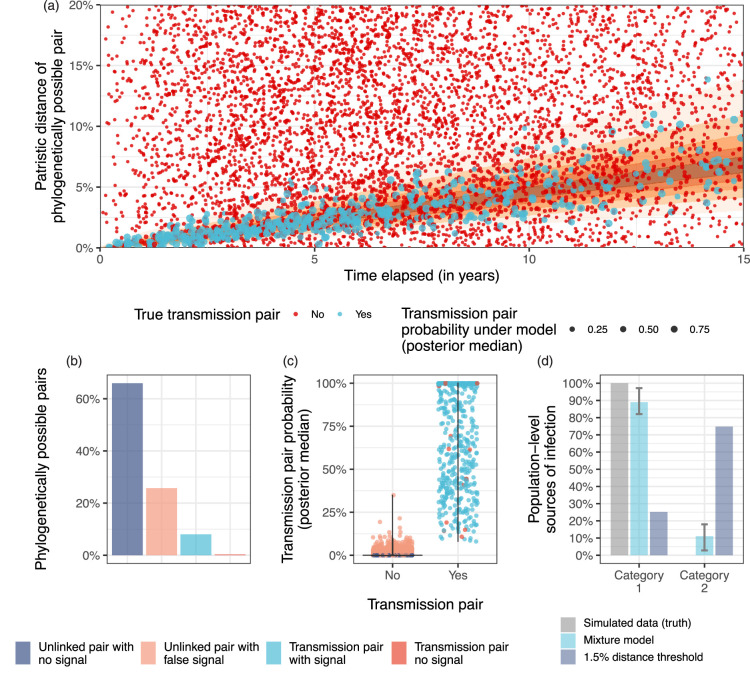
Source attribution with the covariate Bayesian mixture model (BMM) in the case of many competing phylogenetically possible sources per new case. (a) Patristic distances (number of substitutions per 100 nucleotides) and time elapsed for each phylogenetically possible pair, with true transmission pairs shown in blue and unlinked pairs in red. The size of observations corresponds to their posterior probability of being a true transmission pair inferred from the covariate BMM. (b) The empirical proportion of pairs with and without transmission pair signal (based on 95% quantiles of a molecular clock), for actual transmission pairs and unlinked pairs. (c) The posterior probability of being a transmission pair, split by actual transmission pairs and unlinked pairs. (d) Posterior median estimates of transmission flows and 95% credible intervals under the covariate BMM, compared to using a 1.5% threshold on patristic distances.

We next returned to simulated transmission dynamics under the HPTN071/PopART model that included many more pairs with false transmission pair signal in the BMM signal component. We considered the same binary source attribution problem as before, allocating all actual transmitting partners to have the same population-level characteristic “category 1” and all unlinked, but phylogenetically possible sources to “category 2.” In this simulation, 26% of pairs were unlinked with false transmission pair signal, and 0.3% of pairs had no transmission pair signal despite being linked transmission pairs (Figure 5(b)). Fitting the vanilla BMM (13), we estimated that 45% (43%–47%) of transmissions were attributed to category 1, implying an MAE of 55% (52%–57%). For comparison, when we classified phylogenetically likely transmission pairs based on patristic distances <1.5%, we estimated that 25% of transmissions were attributed to category 1, and the MAE was 75%. These simulation results indicate that the error in phylogenetic source attribution can be very large with either a standard classification approach or the vanilla BMM. In particular, the large estimation error is associated with situations in which the phylogenetically possible pairs that are falsely classified as transmission pairs outnumber those pairs that are correctly classified.

To improve on this poor estimation accuracy, we next considered the covariate BMM (16) that included as a predictor of the BMM mixing weights (16c) the binary covariates: “category 1” and “category 2.” In other words, we added features that make the classification task perfectly identifiable upon correct parameterization of the BMM. Fitting the model, we found that nearly all previous pairs with false transmission pair signal were now estimated to have a low posterior probability of being a transmission pair (Figure 5(c)) and 89% [82%–97%] of transmission events were attributed to category 1, implying an MAE of 11% (3%–18%) (Figure 5(d)). These simulations indicate that the covariate BMM can accurately estimate population-level drivers of transmission even when many pairs exhibit false transmission pair signal, provided that additional covariate data are available that perfectly separate transmission pairs from unlinked pairs.

**Figure 6.**
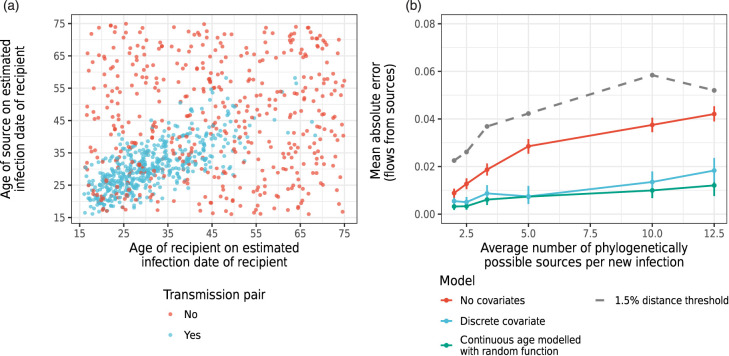
Performance of three models, benchmarked against inference through a fixed genetic distance threshold. (a) Structure of the simulated ages of sources and recipients in true transmission pairs and unlinked pairs. (b) Mean absolute error (MAE) in the estimated transmission flows from each source category (age group) under the three models. MAE for flows by estimating sources with a 1.5% patristic distance threshold. We note that the MAE cannot be directly compared to that in Figure 4 because there are more strata from which we estimate the sources.

More realistically, we considered a (non-binary) source attribution scenario in which simulated transmission events were associated with similarity in the age of newly acquired cases and their transmitting partners. Data were simulated so that age similarity did not perfectly separate transmission pairs from unlinked pairs (Figure 6(a)). For the actual transmission pairs, we simulated the age of the sources using a truncated log-normal distribution between 16 and 75 with a mean of 30 years and simulated the age of the recipients in each pair using the age of their source as a mean to generate correlated ages. We simulated the ages of the unlinked pairs uniformly across the same age range. We included the ages of the cases and their phylogenetically possible sources as independent predictors of the mixing weights of the covariate BMM and sought to recover the age profile of the sources of transmission in the simulation by 5-year age groups. We performed several experiments, keeping the average number of phylogenetically possible sources per incident cases in each experiment fixed at 2, 2.5, 3.3, 5, 10, 12.5, to represent scenarios with minimal to pervasive false transmission pair signal (Figure 6(b)). We also compared source attribution using a 1.5% patristic distance threshold to classify phylogenetically likely transmission pairs, the vanilla BMM, and an HSGP random function BMM (17) in which the mixing weights were modeled through a 2D random function on the age of the newly acquired case and their phylogenetically possible source. Table 1 shows that the HSGP random function BMM had consistently the lowest MAE, and was 0.3% [0.2%–0.5%] for an average of two phylogenetically possible sources per newly acquired case, increasing to 0.7% [0.4%–1.1%] for an average of five phylogenetically possible sources per newly acquired case, and 1.2% [0.8%–1.7%] for an average of 12.5 phylogenetically possible sources per case. Note that the MAE cannot be directly compared with earlier results since we estimated the sources across more strata.

**Table 1.** Accuracy in phylogenetic source attribution by 5-year age bands on simulated data with substantial false transmission pair signal.

	Mean absolute error (%)*			
Average number	Pairs classified			
of sources per	by patristic			HSGP random
recipient	distances <1.5 %	Vanilla BMM	Covariate BMM †	function BMM ‡
2	2.3%	0.9% [0.7%–1.1%]	0.5% [0.3%–0.8%]	0.3% [0.2%–0.5%]
2.5	2.6%	1.3% [1.1%–1.5%]	0.5% [0.3%–0.7%]	0.3% [0.2%–0.5%]
3.3	3.7%	1.9% [1.6%–2.1%]	0.9% [0.6%–1.2%]	0.6% [0.4%–0.9%]
5	4.2%	2.9% [2.5%–3.2%]	0.7% [0.4%–1.2%]	0.7% [0.4%–1.1%]
10	5.8%	3.7% [3.4%–4.1%]	1.4% [1%–1.8%]	1% [0.7%–1.4%]
12.5	5.2%	4.2% [3.9%–4.5%]	1.8% [1.4%–2.4%]	1.2% [0.8%–1.7%]

BMM: Bayesian mixture model; HSGP: Hilbert-space Gaussian process.

*Mean absolute error reports the estimation error in the attributed source categories from each model.

† Covariate BMM includes categorical information on both the source and recipient at the time of infection of the recipient.

‡ HSGP BMM incorporates a continuous variable (e.g. 1-year age) on both the source and recipient at the time of infection of the recipient.

### 3.5. Age-specific drivers of transmission in Amsterdam MSM transmission chains

**Figure 7.**
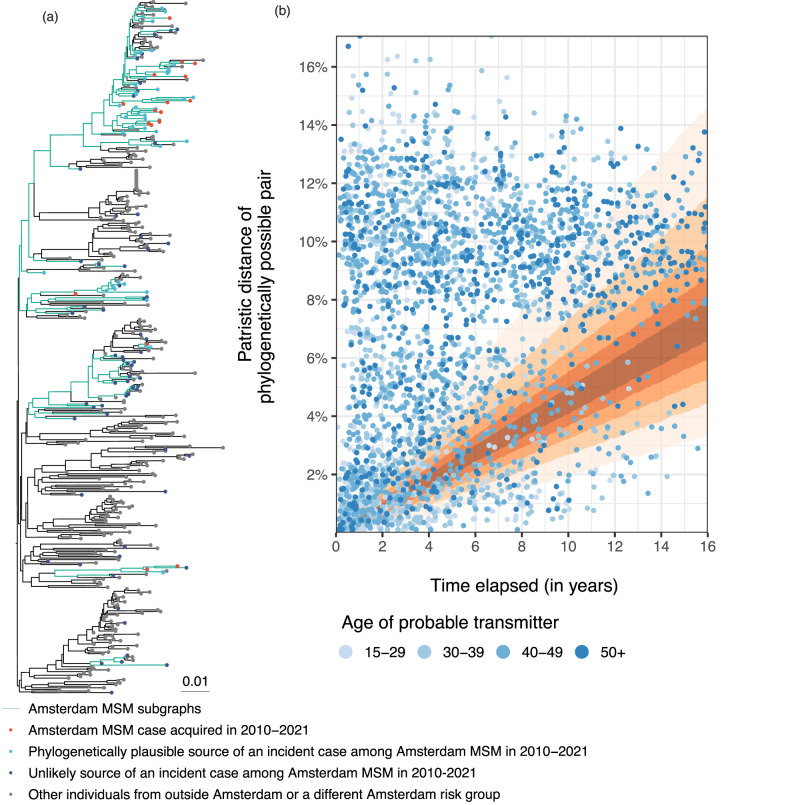
Phylogenetic data from Amsterdam men who have sex with men (MSM). (a) Clade of subtype B among Amsterdam MSM. A clade is a subset of the phylogenetic tree including all descendents of an ancestral lineage. MSM subgraphs are identified by colored branches. Members of MSM subgraphs with an infection date since 2010 have red tips; members who are a phylogenetically possible source for these recipients have light blue tips; members who are neither a source nor a recipient have dark blue tips. (b) Patristic distances and estimated time elapsed for phylogenetically possible transmission pairs of Amsterdam MSM.

Finally, we illustrate our method on data from HIV transmission chains among Amsterdam MSM. Data on people living with HIV in the Netherlands were obtained from the ATHENA observational cohort, comprising demographic, clinical, and viral sequence data for diagnosed individuals until 17 January 2022.^37^ Amsterdam residents were geolocated using residential postal codes at enrollment, or at a registration update. Longitudinal CD4 and viral load measurements enabled us to estimate HIV infection times with a Bayesian model trained on data from 19,788 seroconverters with known date of last negative test from the CASCADE collaboration.^38,39^ We used posterior median infection time estimates to calculate the time elapsed (6). HIV phylogenies and phylogenetic transmission chains for the major subtypes and CRFs among Amsterdam MSM were reconstructed in the context of 1321 additional HIV sequences from Amsterdam residents in other HIV risk groups, 7119 from the rest of the Netherlands and 12,821 international sequences using *FastTree* and *phyloscanner* (see Section 2). Figure 7(a) illustrates the resulting data for a large clade of all subtype B samples.

Our primary aim was to quantify age-specific population-level sources of transmissions among Amsterdam MSM between 2010 and 2021. In total, there were 1335 new HIV diagnoses among Amsterdam MSM with an estimated infection date during the study period, and 840 had an HIV sequence of one of the predominant HIV-1 subtypes or CRFs B, C, 01AE, A1, 02AG, D, G, F1, 06cpx available. Of these, 524 Amsterdam MSM formed a phylogeographically distinct transmission chain of more than one member, which we focus on in our analysis. The remainder were considered to have acquired HIV from a non-Amsterdam MSM source, or unobserved Amsterdam MSM. For these, we identified 3033 potential sources among Amsterdam MSM with an estimated infection date that preceded that of the new case, which formed 1,372,332 potential transmission pairs. Of these, we excluded 65,104 (4.74%) pairs with potential sources who were deceased prior to the infection date, 16,934 (1.23%) with potential sources who migrated to the Netherlands after the infection date, 906,756 (66.07%) with potential sources who were estimated to have suppressed and thus untransmittable virus at the infection date, 13,140 (0.96%) with potential sources who had an implausibly long time elapsed, and 367,574 (26.78%) with potential sources who were not part of the same phylogenetically reconstructed transmission chain. Seven of the remaining phylogenetically possible pairs had a distance of zero (i.e. their sequences were identical). Examining metadata for these pairs suggested these were distinct individuals, so we reset their patristic distance to one mutation across the length of the alignment (0.077% substitution rate), but carried out a sensitivity analysis to assess the impact of omitting these pairs (see Supplemental Material S5.3).

In total, this left 409 sampled new Amsterdam MSM cases with at least one sampled Amsterdam MSM as a phylogenetically possible source. Each new case had a median of 3 and an average of 6.9 phylogenetically possible sources, and there were a total of 2824 phylogenetically possible transmission pairs. The median time elapsed was 4.17 years, and thus we expected many phylogenetically possible transmission pairs to have patristic distances above a patristic distance threshold of 1.5% since the dates at which viral sequences could be obtained were long after the estimated infection time. Many phylogenetically possible pairs had patristic distances outside of the posterior predictive distribution of the clock model, suggesting these are unlikely to be true transmission pairs given their incompatibility with the molecular clock (Figure 7(b)). A small proportion of pairs had a small patristic distance relative to their time elapsed. These could be true transmission pairs, with individual-level infection date uncertainty resulting in an error in the estimated time elapsed, or could be unlinked pairs from the same phylogenetic cluster, with an intermediary person between them in the unobserved transmission chain. We fitted the HSGP random function BMM (17), with the mixing weights specified through a 2D random function on the age of the recipient and the source at the estimated infection time of each phylogenetically possible transmission pair. The model converged and mixed well with no divergences and a runtime of 59 min on a 2020 MacBook Pro (Supplemental Figures S4 and S5). This model had an MAE of <1.2 % in simulations configured to the same average number of pairs per incident case (Supplemental Table S2), and out of all models considered, was the model with the highest expected log posterior density (Supplemental Table S3). Only two phylogenetically possible transmission pairs were associated with posterior transmission probabilities above 95% but even for these we cannot rule out unsampled intermediates or unsampled sources, and therefore cannot interpret the corresponding phylogenetically possible sources with any certainty as the actual transmitting partner. We thus only considered the phylogenetically possible sources as weighted by their posterior transmission pair probabilities in aggregate, and focus on their population-level age characteristics. We explored and validated alternative background distributions by comparing the inferred average mixing weights across pairs with the expected proportion of true transmission pairs, which is given by the ratio of incident cases to all possible pairs. Assuming a uniform background distribution, we successfully recovered a posterior mixing weight consistent with the expected proportion of transmission pairs, while other candidate models over-estimated the proportion of transmission pairs (see Supplemental Material S5.4).

**Figure 8.**
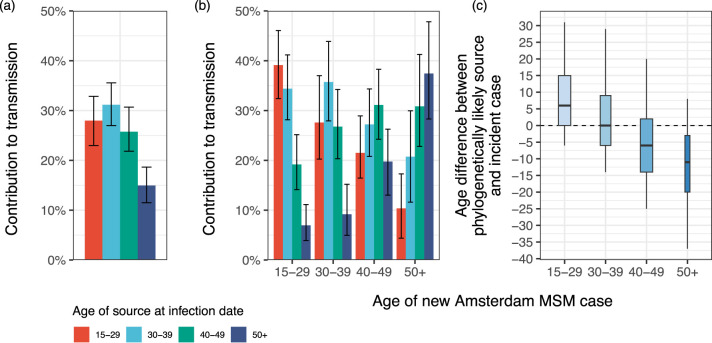
Estimated age of Amsterdam men who have sex with men (MSM) sources of transmission in Amsterdam MSM who acquired infection in 2010–2021. (a) Overall contribution of age groups to transmissions within Amsterdam MSM transmission networks in 2010–2021 (bar: posterior median; errorbar: 95% posterior credible intervals). (b) Contribution of age groups to transmissions within each recipient age band of 15–29 years, 30–39 years, 40–49 years, and 50 years and above (bar: posterior median; errorbar: 95% posterior credible intervals). (c) Estimated age difference between the sources and recipients in likely transmission pairs, with a positive age difference indicating older likely sources. The thick line within boxplots indicates the posterior median age difference, the box the posterior interquartile range, the whiskers the 2.5% and 97.5% posterior quantiles, and the widths of the boxplots are proportional to number of likely pairs who have a recipient in each recipient age band of 15–29 years, 30–39 years, 40–49 years, and 50 years and above.

Infection times were bootstrap sampled 50 times and models were refitted to account for uncertainty in infection time estimates. We found that transmissions originated from all age groups among Amsterdam MSM in 2010–2021, with an estimated 28% [23%–33%] from 15 to 29-year-olds, 31% [27%–36%] from 30 to 39-year-olds, 26% [22%–31%] from 40 to 49-year-olds, and 15% [12%–19%] from MSM aged 50 and above (Figure 8(a)). Stratifying by age of recipients, we found that most incident cases had a source within the same age band, but not strongly so. For all age groups, more than half of incident cases originated from sources that were either older or younger. For example, for Amsterdam MSM aged 15–29, an estimated 39% [32%–46%] of incident cases originated from 15 to 29-year-olds, 34% [28%–41%] from 30 to 39-year-olds, 19% [14%–25%] from 40 to 49-year-olds, and 7% [4%–11%] from MSM aged 50 and above (Figure 8(b)). Considering transmissions by age of the recipients, our data indicated that the age structure of transmission sources is shifting from older sources to incident Amsterdam MSM aged 15–29 to younger sources to incident Amsterdam MSM aged 50 and above. To examine this further, we calculated the age gap between the age of the phylogenetically possible source and the age of the new case at the likely time of the infection event and weighted these age gaps by the posterior probability that the pair represents an transmission event (Supplemental Material S6). We estimate that 15–29-year-old Amsterdam MSM had sources of transmissions who were on average 6 years older (posterior interquartile range [IQR] 0–15 years) while 30–39-year-olds had sources on average the same age (IQR 6 years younger to 9 years older), 40–49-year-olds had sources on average 6 years younger (IQR 14 years younger to 2 years older), and Amsterdam MSM aged over 50 had sources that were on average 11 years younger (IQR 3 to 20 years younger).

## 4. Discussion

We present a novel approach to phylogenetic source attribution that combines molecular genetic distances between sampled pathogen sequences with time elapsed in a BMM that has a population-level molecular clock as its signal component. This statistical approach to source attribution is now becoming increasingly possible as various methods are now available for estimating infection times from clinical and demographic data as we have used here,^70,71^ but also from deep-sequence data,^72–75^ the number of ambiguous nucleotide mutations in consensus sequences,^76–79^ and combinations of biomarker and pathogen sequence data.^80,81^ The main additional information needed to leverage these time since infection estimates are the evolutionary clock model that underpins the signal component of the BMM. We focused on developing the approach for HIV transmission dynamics because a previous study^58^ sequenced a large number of pathogens from a known transmission chain, enabling us to use a large dataset of over 2800 sequence pairs to construct the signal component. Similar approaches, or even using molecular clocks parameterized as part of large-scale phylogenetic analyses,^82^ could be explored for a wide range of other pathogens that mutate sufficiently rapidly relative to the time scale of transmission dynamics.^83,84^

A central insight from our investigation is that the signal derived from combining molecular genetic distance measures with time elapsed remains ambiguous, and there is as in previous research^26,61,85^ no way to identify the source of transmission with certainty. Simulations based on the PopART-IBM HIV transmission model demonstrate that the large majority of data points falling into the BMM signal component correspond to unlinked pairs of individuals, and thus exhibit false signal that can introduce significant bias to population-level source attribution inferences. Additional features are necessary to reduce misclassification rates on the true, unknown transmission status between individuals in phylogenetically possible transmission pairs to a tolerable level for source attribution. These features may vary between pathogens and also between localized epidemics. For example, HIV suppression status will be most informative for estimating HIV transmission sources in populations with well-established treatment programs and regular monitoring of ART response. For infectious diseases that can be cured, clinical data on cleared infections could similarly be used to ascertain when individuals were uninfectious. Diagnosis dates can be highly informative for pathogens whose serial intervals to the diagnosis date of the next infection are short.^86–88^ Mortality data can be useful in narrowing down the potential sources of infection for life-long infectious diseases, and mobility data can be useful in excluding potential transmission sources where populations are highly mobile or suffer from displacement or armed conflict. From a methodological angle, these considerations imply that the vanilla BMM (13) is unlikely to provide accurate source attribution inferences, and we recommend using the covariate or random function BMMs (16) and (17), or mixtures of both. Other phylodynamic approaches based on inferring transmission trees from genomic data and infection times can be applied^89^ using reversible jump Markov chain Monte Carlo which accounts for unsampled individuals. However, these are best suited to large, rapidly spreading outbreaks, whilst in the case of HIV there are generally many small concurrent transmission chains consisting of few individuals.

Here, we used the random function BMM to investigate the age groups that underpin the continued spread of HIV within Amsterdam MSM transmission chains, excluding those for whom phylogenetic data indicate that they were likely infected by an individual not resident in Amsterdam. We found that no single age group drove transmission among MSM in Amsterdam between 2010 and 2021, though 30–39-year-olds contributed the highest proportion of transmissions. Analysis of deep-sequence data from six European countries participating in the BEEHIVE study, also identified MSM aged 30–39 to be the most likely source of infections across all age groups among phylogenetically linked pairs.^90^ We found limited evidence of assortative mixing across all age groups, with the majority of infections in each of the recipient age bands 15–29 years, 30–39 years, 40–49 years, and 50 years and above originating from outside these age groups, respectively. Behavioural survey data collected from MSM in Amsterdam in 2008–2009 found disassortive mixing by age, particularly between casual partners,^91^ and phylogenetic evidence from MSM in Switzerland suggested an average overall age gap of 9 years between inferred transmission pairs.^92^ We estimated age gaps of five years between MSM under 30 and their likely sources, and MSM over 40 were between five and 12 years younger than their likely sources. Age of sources has previously been found to depend on the age of the recipient at the time of infection, with MSM under 30 in the European BEEHIVE study estimated to have a source on average 6 years older, and over 40 were estimated to have a source 8 years younger,^90^ cohesive with our findings. Other studies in the United States, focused on HIV transmission to young MSM, have also identified age gaps between recipients and older sources.^93,94^ However, a study among Tennessee MSM found evidence from phylogenetic analysis of more transmission between young MSM than from older partners.^95^ Incorporating other covariates, White MSM have been estimated to have larger age gaps between young recipients and their sources than other races and ethnicities across the US,^67^ so age gaps may also be driven by population demographics. We did not quantify this in our study, however, over 40% of new diagnoses in Amsterdam MSM are among individuals with a migration background,^96^ suggesting age gaps may be heterogeneous depending on the ethnicity of incident cases.

Our statistical approach has a number of important limitations. First, underpinning the model are the infection dates of individuals, which are usually unknown but can be estimated. Existing methods for estimating infection time have in general been shown to suffer from individual-level uncertainty.^38,75,81^ This is carried through to the time elapsed, which can lead to false transmission pair signal among truly unlinked pairs or lack of signal for actual transmission pairs, and thereby distort population-level flow estimates. However, accounting for infection time uncertainty in the application found no indications of substantial bias, though uncertainty intervals were larger. Second, we do not adjust for incomplete sampling in the model, meaning the true source for an incident case may not be among the phylogenetically possible sources. In practical applications, for many pathogens and populations, it is likely that a proportion of individuals do not have a sequence, due to being undiagnosed, or for reasons of study enrollment and consent.^20^ For example, we were unable to fit the model to data from heterosexual phylogenies from Amsterdam, due to small sample sizes and low sequence coverage. Approaches have been developed for assessing whether datasets have sufficient samples to identify truly linked transmission pairs,^97^ and for accounting for incomplete sampling in source attribution, which goes beyond the scope of this study but could be incorporated in practice.^98,99^ Thirdly, we focus on developing a parsimonious clock model to nest within the mixture model. There is limited data available for pairs with a time elapsed of less than one year and above 15 years to train the model; as a result, there is a large uncertainty associated with inferences made for individuals with values outside this range. In addition, we consider a linear trend in the within-host evolutionary rate over time, though it is possible the rate decreases over a prolonged period.^100^ For reasons of parsimony, we also assume a uniform background distribution in the mixture model. The background distribution may depend on the specific application, and simulations could be used to select an appropriate distribution. Finally, we have demonstrated the application of this method to estimating sources of HIV. However, it could be applied to other fast-evolving pathogens such as Hepatitis C and Ebola, if similar data from confirmed transmission pairs, or existing estimates of their evolutionary rate, are available.^101,102^ Other predictors, with existing evidence of their association with known transmitters, may be used in place of age to inform the BMM mixture weights, and population-level transmission flows may be summarized by other covariates of interest.^103^

In summary, this paper develops a mixture model framework for incorporating time since infection estimates and pathogen genomic data to estimate population-level sources of pathogen spread. We find that time since infection estimates are informative about characterizing transmission sources, both through reducing the number of potential sources for each new infection case, and by providing the data needed to interpret pathogen genomic data in the context of the signal derived from pathogen-specific molecular clocks. We also find that individual-level sources of transmission cannot be identified even with additional time since infection estimates, and demonstrate that false transmission pair signal is pervasive in realistic simulations of HIV spread as well as real-world data from Amsterdam. This prompted us to take a Bayesian approach that integrates uncertainty in individual-level class labels and estimates the relevance of additional covariates for source attribution through modeling generalized linear predictors of the mixture model mixing weights. The model has been principally developed to characterize HIV transmission flows, but is readily applicable to source attribution of other pathogens providing pathogen-specific molecular clocks can be specified.

## Supplemental Material

sj-pdf-1-smm-10.1177_09622802241309750 - Supplemental material for Bayesian mixture models for phylogenetic source attribution from consensus sequences and time since infection estimates Supplemental material, sj-pdf-1-smm-10.1177_09622802241309750 for Bayesian mixture models for phylogenetic source attribution from consensus sequences and time since infection estimates by Alexandra Blenkinsop, Lysandros Sofocleous, Francesco Di Lauro, Evangelia Georgia Kostaki, Ard van Sighem, Daniela Bezemer, Thijs van de Laar, Peter Reiss, Godelieve de Bree, Nikos Pantazis, Oliver Ratmann and on behalf of the HIV Transmission Elimination Amsterdam (H-TEAM) Consortium in Medical Research

## References

[1] Hill V Ruis C Bajaj S , et al. Progress and challenges in virus genomic epidemiology. Trends Parasitol 2021; 37: 1038–1049. 34620561 10.1016/j.pt.2021.08.007

[2] Faria NR Mellan TA Whittaker C , et al. Genomics and epidemiology of the p.1 SARS-CoV-2 lineage in Manaus, Brazil. Science 2021; 372: 815–821. 33853970 10.1126/science.abh2644 PMC8139423

[3] Waddington C Carey ME Boinett CJ , et al. Exploiting genomics to mitigate the public health impact of antimicrobial resistance. Genome Med 2022; 14: 15. 35172877 10.1186/s13073-022-01020-2 PMC8849018

[4] Dudas G Carvalho LM Bedford T , et al. Virus genomes reveal factors that spread and sustained the Ebola epidemic. Nature 2017; 544: 309–315. 28405027 10.1038/nature22040 PMC5712493

[5] Little SJ Pond SLK Anderson CM , et al. Using HIV networks to inform real time prevention interventions. PLoS ONE 2014; 9: e98443. 10.1371/journal.pone.0098443 PMC4047027 24901437

[6] Grabowski MK Redd AD . Molecular tools for studying HIV transmission in sexual networks. Curr Opin HIV AIDS 2014; 9: 126. 24384502 10.1097/COH.0000000000000040 PMC4109889

[7] Karthikeyan S Levy JI Hoff PD , et al. Wastewater sequencing reveals early cryptic SARS-CoV-2 variant transmission. Nature 2022; 609: 101–108. 35798029 10.1038/s41586-022-05049-6 PMC9433318

[8] Crits-Christoph A Kantor RS Olm MR , et al. Genome sequencing of sewage detects regionally prevalent SARS-CoV-2 variants. mBio 2021; 12. DOI: 10.1128/mbio.02703-20. PMC7845645 33468686

[9] Arenas M . Trends in substitution models of molecular evolution. Front Genet 2015; 6: 319. 26579193 10.3389/fgene.2015.00319 PMC4620419

[10] Wymant C Hall M Ratmann O , et al. PHYLOSCANNER: Inferring transmission from within- and between-host pathogen genetic diversity. Mol Biol Evol 2018; 35: 719–733. 29186559 10.1093/molbev/msx304 PMC5850600

[11] Viana R Moyo S Amoako DG , et al. Rapid epidemic expansion of the SARS-CoV-2 Omicron variant in Southern Africa. Nature 2022; 603: 679–686. 35042229 10.1038/s41586-022-04411-y PMC8942855

[12] Volz E Mishra S Chand M , et al. Assessing transmissibility of SARS-CoV-2 lineage b.1.1.7 in England. Nature 2021; 593: 266–269. 33767447 10.1038/s41586-021-03470-x

[13] Mate SE Kugelman JR Nyenswah TG , et al. Molecular evidence of sexual transmission of ebola virus. N Engl J Med 2015; 373: 2448–2454. 26465384 10.1056/NEJMoa1509773 PMC4711355

[14] Brenner B Ibanescu RI Hardy I , et al. Genotypic and phylogenetic insights on prevention of the spread of HIV-1 and drug resistance in “real-world” settings. Viruses 2017; 10: 10. 29283390 10.3390/v10010010 PMC5795423

[15] Aanensen DM Carlos CC Donado-Godoy P , et al. Implementing whole-genome sequencing for ongoing surveillance of antimicrobial resistance: Exemplifying insights into klebsiella pneumoniae. Clin Infect Dis 2021; 73: S255–S257. 10.1093/cid/ciab795 PMC8634455 34850830

[16] Volz EM Ionides E Romero-Severson EO , et al. HIV-1 transmission during early infection in men who have sex with men: A phylodynamic analysis. PLoS Med 2013; 10: e1001568. 10.1371/journal.pmed.1001568 PMC3858227 24339751

[17] Ratmann O van Sighem A Bezemer D , et al. Sources of HIV infection among men having sex with men and implications for prevention. Sci Transl Med 2016; 8: 320ra2. 10.1126/scitranslmed.aad1863 PMC4902123 26738795

[18] Chan SK Thornton LR Chronister KJ , et al. Likely female-to-female sexual transmission of HIV—texas, 2012. Morb Mortal Wkly Rep 2014; 63: 209–212. PMC5779339 24622284

[19] Stadler T Bonhoeffer S . Uncovering epidemiological dynamics in heterogeneous host populations using phylogenetic methods. Philos Trans R Soc B: Biol Sci 2013; 368: 20120198. 10.1098/rstb.2012.0198 PMC3678323 23382421

[20] Volz EM Frost SDW . Inferring the source of transmission with phylogenetic data. PLoS Comput Biol 2013; 9: e1003397. 10.1371/journal.pcbi.1003397 PMC3868546 24367249

[21] Colijn C Gardy J . Phylogenetic tree shapes resolve disease transmission patterns. Evol Med Public Health 2014; 2014: 96–108. 24916411 10.1093/emph/eou018 PMC4097963

[22] Müller NF Wüthrich D Goldman N , et al. Characterising the epidemic spread of influenza A/H3N2 within a city through phylogenetics. PLoS Pathog 2020; 16: e1008984. 10.1371/journal.ppat.1008984 PMC7676729 33211775

[23] Bachmann N Kusejko K Nguyen H , et al. Phylogenetic cluster analysis identifies virological and behavioral drivers of human immunodeficiency virus transmission in men who have sex with men. Clin Infect Dis 2020; 72: 2175–2183. 10.1093/cid/ciaa411 32300807

[24] Labarile M Loosli T Zeeb M , et al. Quantifying and predicting ongoing human immunodeficiency virus type 1 transmission dynamics in Switzerland using a distance-based clustering approach. J Infect Dis 2022; 227: 554–564. 10.1093/infdis/jiac457 36433831

[25] Fisher M Pao D Brown AE , et al. Determinants of HIV-1 transmission in men who have sex with men: A combined clinical, epidemiological and phylogenetic approach. AIDS 2010; 24: 1739–1747. 20588173 10.1097/QAD.0b013e32833ac9e6

[26] Reichmuth M Chaudron S Bachmann N , et al. Using longitudinally sampled viral nucleotide sequences to characterize the drivers of HIV-1 transmission. HIV Med 2020; 22: 346–359. 33368946 10.1111/hiv.13030

[27] Romero-Severson EO Bulla I Leitner T . Phylogenetically resolving epidemiologic linkage. Proc Natl Acad Sci USA 2016; 113: 2690–2695. 26903617 10.1073/pnas.1522930113 PMC4791024

[28] Magosi LE Zhang Y Golubchik T , et al. Deep-sequence phylogenetics to quantify patterns of HIV transmission in the context of a universal testing and treatment trial – BCPP/ya tsie trial. eLife 2022; 11: e72657. 10.7554/eLife.72657 PMC8912920 35229714

[29] Xi X Spencer SEF Hall M , et al. Inferring the sources of HIV infection in Africa from deep-sequence data with semi-parametric Bayesian poisson flow models. J R Stat Soc: Ser C (Appl Stat) 2022; 71: 517–540.

[30] Grabowski MK Herbeck JT Poon AFY . Genetic cluster analysis for HIV prevention. Curr HIV/AIDS Rep 2018; 15: 182–189. 29460226 10.1007/s11904-018-0384-1 PMC5882762

[31] Stimson J Gardy J Mathema B , et al. Beyond the SNP threshold: Identifying outbreak clusters using inferred transmissions. Mol Biol Evol 2019; 36: 587–603. 30690464 10.1093/molbev/msy242 PMC6389316

[32] Silva DD Peters J Cole K , et al. Whole-genome sequencing to determine transmission of Neisseria gonorrhoeae: An observational study. Lancet Infect Dis 2016; 16: 1295–1303. 27427203 10.1016/S1473-3099(16)30157-8 PMC5086424

[33] Kemp SA Collier DA Datir RP , et al. SARS-CoV-2 evolution during treatment of chronic infection. Nature 2021; 592: 277–282. 33545711 10.1038/s41586-021-03291-y PMC7610568

[34] McLachlan GJ Peel D . Finite mixture models (Wiley series in probability and statistics). New York: Wiley, 2000.

[35] Rasmussen C . The infinite Gaussian mixture model. In: Solla S, Leen T, and Müller K (eds) *Advances in neural information processing systems*, vol. 12. MIT Press, 1999. https://papers.nips.cc/paper_files/paper/1999/hash/97d98119037c5b8a9663cb21fb8ebf47-Abstract.html.

[36] Rodriguez A Dunson DB Taylor J . Bayesian hierarchically weighted finite mixture models for samples of distributions. Biostatistics 2008; 10: 155–171. 18708650 10.1093/biostatistics/kxn024 PMC2733158

[37] Boender TS Smit C Sighem Av , et al. AIDS therapy evaluation in the Netherlands (ATHENA) national observational HIV cohort: Cohort profile. BMJ Open 2018; 8 e022516. 10.1136/bmjopen-2018-022516 PMC6169757 30249631

[38] Pantazis N Thomadakis C Del Amo J , et al. Determining the likely place of HIV acquisition for migrants in Europe combining subject-specific information and biomarkers data. Stat Methods Med Res 2019; 28: 1979–1997. 29233073 10.1177/0962280217746437

[39] Blenkinsop A Monod M Sighem Av , et al. Estimating the potential to prevent locally acquired HIV infections in a UNAIDS fast-track city, Amsterdam. eLife 2022; 11: e76487. 10.7554/eLife.76487 PMC9545569 35920649

[40] Zhao B Qiu Y Song W , et al. Undiagnosed HIV infections may drive HIV transmission in the era of “treat all”: A deep-sampling molecular network study in northeast china during 2016 to 2019. Viruses 2022; 14: 1895. 36146701 10.3390/v14091895 PMC9502473

[41] Cauchemez S Bhattarai A Marchbanks TL , et al. Role of social networks in shaping disease transmission during a community outbreak of 2009 H1N1 pandemic influenza. Proc Natl Acad Sci USA 2011; 108: 2825–2830. 21282645 10.1073/pnas.1008895108 PMC3041067

[42] Pantaleo G Graziosi C Fauci AS . The immunopathogenesis of human immunodeficiency virus infection. New Engl J Med 2010; 328: 327–335. 10.1056/NEJM199302043280508 8093551

[43] Ratmann O Kagaayi J Hall M , et al. Quantifying HIV transmission flow between high-prevalence hotspots and surrounding communities: A population-based study in Rakai, Uganda. Lancet HIV 2020; 7: e173–e183. 10.1016/S2352-3018(19)30378-9 PMC7167508 31953184

[44] Adam DC Martın-Sánchez M Gu H , et al. Risk of within-hotel transmission of SARS-CoV-2 during on-arrival quarantine in Hong Kong: An epidemiological and phylogenomic investigation. Lancet Reg Health - West Pac 2023; 100678. 36643735 10.1016/j.lanwpc.2022.100678 PMC9825110

[45] Rodger AJ Cambiano V Bruun T , et al. Sexual activity without condoms and risk of HIV transmission in serodifferent couples when the HIV-positive partner is using suppressive antiretroviral therapy. JAMA 2016; 316: 171. 27404185 10.1001/jama.2016.5148

[46] Bavinton BR Pinto AN Phanuphak N , et al. Viral suppression and HIV transmission in serodiscordant male couples: An international, prospective, observational, cohort study. Lancet HIV 2018; 5: e438–e447. 10.1016/S2352-3018(18)30132-2 30025681

[47] Bezemer D Jurriaans S Prins M , et al. Declining trend in transmission of drug-resistant HIV-1 in Amsterdam. AIDS 2004; 18: 1571–1577. 15238775 10.1097/01.aids.0000131357.52457.33

[48] Price MN Dehal PS Arkin AP . Fasttree 2 – approximately maximum-likelihood trees for large alignments. PLoS ONE 2010; 5: 1–10. 10.1371/journal.pone.0009490 PMC2835736 20224823

[49] Nguyen LT Schmidt HA von Haeseler A , et al. IQ-TREE: A fast and effective stochastic algorithm for estimating maximum-likelihood phylogenies. Mol Biol Evol 2014; 32: 268–274. 25371430 10.1093/molbev/msu300 PMC4271533

[50] Drummond AJ Rambaut A . BEAST: Bayesian evolutionary analysis by sampling trees. BMC Evol Biol 2007; 7: 214. 17996036 10.1186/1471-2148-7-214 PMC2247476

[51] Lemey P Rambaut A Drummond AJ , et al. Bayesian phylogeography finds its roots. PLoS Comput Biol 2009; 5: e1000520. 10.1371/journal.pcbi.1000520 PMC2740835 19779555

[52] Maio ND Wu CH O’Reilly KM , et al. New routes to phylogeography: A Bayesian structured coalescent approximation. PLoS Genet 2015; 11: e1005421. 10.1371/journal.pgen.1005421 PMC4534465 26267488

[53] Müller NF Rasmussen DA Stadler T . The structured coalescent and its approximations. Mol Biol Evol 2017; 34: 2970–2981. 28666382 10.1093/molbev/msx186 PMC5850743

[54] Bezemer D Blenkinsop A Hall M , et al. Many but small HIV-1 non-B transmission chains in the Netherlands. AIDS 2021; 36: 83–94. 10.1097/QAD.0000000000003074 PMC8655833 34618753

[55] Jombart T Balloux F Dray S . adephylo: New tools for investigating the phylogenetic signal in biological traits. Bioinformatics 2010; 26: 1907–1909. 20525823 10.1093/bioinformatics/btq292

[56] Leitner T Albert J . The molecular clock of HIV-1 unveiled through analysis of a known transmission history. Proc Natl Acad Sci USA 1999; 96: 10752–10757. 10485898 10.1073/pnas.96.19.10752 PMC17955

[57] Lemey P Derdelinckx I Rambaut A , et al. Molecular footprint of drug-selective pressure in a human immunodeficiency virus transmission chain. J Virol 2005; 79: 11981–11989. 16140774 10.1128/JVI.79.18.11981-11989.2005 PMC1212611

[58] Vrancken B Rambaut A Suchard MA , et al. The genealogical population dynamics of HIV-1 in a large transmission chain: Bridging within and among host evolutionary rates. PLoS Comput Biol 2014; 10: e1003505. 10.1371/journal.pcbi.1003505 PMC3974631 24699231

[59] Higgins JPT Thompson SG Spiegelhalter DJ . A re-evaluation of random-effects meta-analysis. J R Stat Soc: Ser A (Stat Soc) 2009; 172: 137–159. 10.1111/j.1467-985X.2008.00552.x PMC2667312 19381330

[60] Alizon S Fraser C . Within-host and between-host evolutionary rates across the HIV-1 genome. Retrovirology 2013; 10: 49. 23639104 10.1186/1742-4690-10-49 PMC3685529

[61] Pillay D Rambaut A Geretti AM , et al. HIV phylogenetics. BMJ 2007; 335: 460–461. 17823148 10.1136/bmj.39315.398843.BE PMC1971185

[62] Riutort-Mayol G Bürkner PC Andersen MR , et al. Practical Hilbert space approximate Bayesian Gaussian processes for probabilistic programming. Stat Comput 2023; 33: 17.

[63] Dan S Chen Y Chen Y , et al. Estimating fine age structure and time trends in human contact patterns from coarse contact data: The Bayesian rate consistency model. PLoS Comput Biol 2023; 19: e1011191. 10.1371/journal.pcbi.1011191 PMC10270591 37276210

[64] Pickles M Cori A Probert WJM , et al. Popart-ibm, a highly efficient stochastic individual-based simulation model of generalised HIV epidemics developed in the context of the HPTN 071 (popART) trial. PLoS Comput Biol 2021; 17: 1–21. 10.1371/journal.pcbi.1009301 PMC8478209 34473700

[65] Stamatakis A . Raxml version 8: A tool for phylogenetic analysis and post-analysis of large phylogenies. Bioinformatics 2014; 30: 1312–1313. 24451623 10.1093/bioinformatics/btu033 PMC3998144

[66] Kosakovsky Pond SL Weaver S Leigh Brown AJ , et al. HIV-TRACE (Transmission cluster engine): A tool for large scale molecular epidemiology of HIV-1 and other rapidly evolving pathogens. Mol Biol Evol 2018; 35: 1812–1819. 29401317 10.1093/molbev/msy016 PMC5995201

[67] Whiteside YO Song R Wertheim JO , et al. Molecular analysis allows inference into HIV transmission among young men who have sex with men in the United States. AIDS 2015; 29: 2517–2522. 26558547 10.1097/QAD.0000000000000852 PMC4862399

[68] Wertheim JO Oster AM Hernandez AL , et al. The international dimension of the US HIV transmission network and onward transmission of HIV recently imported into the United States. AIDS Res Hum Retroviruses 2016; 32: 1046–1053. 27105549 10.1089/aid.2015.0272 PMC5067842

[69] Oster AM Wertheim JO Hernandez AL , et al. Using molecular HIV surveillance data to understand transmission between subpopulations in the united states. J Acquir Immune Defic Syndr 2015; 70: 444–451. 26302431 10.1097/QAI.0000000000000809 PMC4878401

[70] Pantazis N Touloumi G Meyer L , et al. The impact of transient combination antiretroviral treatment in early HIV infection on viral suppression and immunologic response in later treatment. AIDS 2016; 30: 879–888. 26636925 10.1097/QAD.0000000000000991 PMC4794189

[71] Lundgren E Romero-Severson E Albert J , et al. Combining biomarker and virus phylogenetic models improves HIV-1 epidemiological source identification. PLoS Comput Biol 2022; 18: e1009741. 10.1371/journal.pcbi.1009741 PMC9455879 36026480

[72] Poon AF McGovern RA Mo T , et al. Dates of HIV infection can be estimated for seroprevalent patients by coalescent analysis of serial next-generation sequencing data. AIDS 2011; 25: 2019–2026. 21832936 10.1097/QAD.0b013e32834b643c

[73] Puller V Neher R Albert J . Estimating time of HIV-1 infection from next-generation sequence diversity. PLoS Comput Biol 2017; 13: e1005775. 10.1371/journal.pcbi.1005775 PMC5638550 28968389

[74] Carlisle LA Turk T Kusejko K , et al. Viral diversity based on next-generation sequencing of HIV-1 provides precise estimates of infection recency and time since infection. J Infect Dis 2019; 220: 254–265. 30835266 10.1093/infdis/jiz094

[75] Golubchik T Abeler-Dörner L Hall M , et al. HIV-phyloTSI: Subtype-independent estimation of time since HIV-1 infection for cross-sectional measures of population incidence using deep sequence data. *medarXiv* 2022. DOI: 10.1101/2022.05.15.22275117. 10.1186/s12859-025-06189-y PMC12351810 40813968

[76] Kouyos RD von Wyl V Yerly S , et al. Ambiguous nucleotide calls from population-based sequencing of HIV-1 are a marker for viral diversity and the age of infection. Clin Infect Dis 2011; 52: 532–539. 21220770 10.1093/cid/ciq164 PMC3060900

[77] Ragonnet-Cronin M Aris-Brosou S Joanisse I , et al. Genetic diversity as a marker for timing infection in HIV-infected patients: Evaluation of a 6-month window and comparison with BED. J Infect Dis 2012; 206: 756–764. 22826337 10.1093/infdis/jis411

[78] Andersson E Shao W Bontell I , et al. Evaluation of sequence ambiguities of the HIV-1 pol gene as a method to identify recent HIV-1 infection in transmitted drug resistance surveys. Infect Genet Evol 2013; 18: 125–131. 23583545 10.1016/j.meegid.2013.03.050 PMC4066879

[79] Lunar MM Lepej SŽ Poljak M . Sequence ambiguity determined from routine pol sequencing is a reliable tool for real-time surveillance of HIV incidence trends. Infect Genet Evol 2019; 69: 146–152. 30682549 10.1016/j.meegid.2019.01.015

[80] Verhofstede C Fransen K Heuvel AVD , et al. Decision tree for accurate infection timing in individuals newly diagnosed with HIV-1 infection. BMC Infect Dis 2017; 17. DOI: 10.1186/s12879-017-2850-6. PMC5708102 29187159

[81] Stirrup OT Dunn DT . Estimation of delay to diagnosis and incidence in HIV using indirect evidence of infection dates. BMC Med Res Methodol 2018; 18: 65. 29945571 10.1186/s12874-018-0522-x PMC6020319

[82] Vijaykrishna D Bahl J Riley S , et al. Evolutionary dynamics and emergence of panzootic H5N1 influenza viruses. PLoS Pathog 2008; 4: 1–10. 10.1371/journal.ppat.1000161 PMC2533123 18818732

[83] Biek R Pybus OG Lloyd-Smith JO , et al. Measurably evolving pathogens in the genomic era. Trends Ecol Evol 2015; 30: 306–313. 25887947 10.1016/j.tree.2015.03.009 PMC4457702

[84] Grenfell BT Pybus OG Gog JR , et al. Unifying the epidemiological and evolutionary dynamics of pathogens. Science 2004; 303: 327–332. 14726583 10.1126/science.1090727

[85] Villabona-Arenas CJ Hué S Baxter JAC , et al. Using phylogenetics to infer HIV-1 transmission direction between known transmission pairs. Proc Natl Acad Sci USA 2022; 119: e2210604119. 10.1073/pnas.2210604119 PMC9499565 36103580

[86] Cauchemez S Boëlle PY Donnelly CA , et al. Real-time estimates in early detection of SARS. Emerg Infect Dis 2012; 12: 110–113. 10.3201/eid1201.050593 PMC3293464 16494726

[87] Cauchemez S Nouvellet P Cori A , et al. Unraveling the drivers of MERS-coV transmission. Proc Natl Acad Sci USA 2016; 113: 9081–9086. 27457935 10.1073/pnas.1519235113 PMC4987807

[88] Lythgoe KA Hall M Ferretti L , et al. SARS-CoV-2 within-host diversity and transmission. Science 2021; 372: eabg0821. 10.1126/science.abg0821 PMC8128293 33688063

[89] Didelot X Fraser C Gardy J , et al. Genomic infectious disease epidemiology in partially sampled and ongoing outbreaks. Mol Biol Evol 2017; 34: 997–1007. 28100788 10.1093/molbev/msw275 PMC5850352

[90] Hall M Wymant C Ratmann O , et al. Age disparities in European HIV transmission pairs uncovered with viral sequence data. In: *Conference on retroviruses and opportunistic infections*, Boston, Massachusetts, 4–7 March 2018, Abstract number: 960. https://www.croiconference.org/abstract/age-disparities-european-hiv-transmission-pairs-uncovered-viral-sequence-data-0/.

[91] Matser A . *Sexually transmitted infections: Unravelling transmission & impact*. PhD thesis, Amsterdam UMC, 2015.

[92] Kusejko K Kadelka C Marzel A , et al. Inferring the age difference in HIV transmission pairs by applying phylogenetic methods on the HIV transmission network of the Swiss HIV cohort study. Virus Evol 2018; 4: vey024. 10.1093/ve/vey024 PMC6143731 30250751

[93] Wolf E Herbeck JT Rompaey SV , et al. Short communication: Phylogenetic evidence of HIV-1 transmission between adult and adolescent men who have sex with men. AIDS Res Hum Retroviruses 2017; 33: 318. 27762596 10.1089/aid.2016.0061 PMC5372772

[94] Hurt CB Matthews DD Calabria MS , et al. Sex with older partners is associated with primary HIV infection among men who have sex with men in North Carolina. J Acquir Immune Defic Syndr 2010; 54: 185. 20057320 10.1097/QAI.0b013e3181c99114 PMC2877753

[95] Dennis AM Volz E Frost AMSD , et al. HIV-1 transmission clustering and phylodynamics highlight the important role of young men who have sex with men. AIDS Res Hum Retroviruses 2018; 34: 879–888. 30027754 10.1089/aid.2018.0039 PMC6204570

[96] Hiv monitoring report 2022: Human immunodeficiency virus (HIV) infection in the Netherlands. Technical report, 2022. https://www.hiv-monitoring.nl/application/files/1816/6851/5357/NL˙HIV˙MONITORING˙REPORT˙2022.pdf.

[97] Wohl S Giles JR Lessler J . Sample size calculation for phylogenetic case linkage. PLoS Comput Biol 2021; 17: e1009182. 10.1371/journal.pcbi.1009182 PMC8284614 34228722

[98] Ratmann O Grabowski MK Hall M , et al. Inferring HIV-1 transmission networks and sources of epidemic spread in Africa with deep-sequence phylogenetic analysis. Nat Commun 2019; 10: 1411. 30926780 10.1038/s41467-019-09139-4 PMC6441045

[99] Hall M Golubchik T Bonsall D , et al. Demographic characteristics of sources of HIV-1 transmission in the era of test and treat. *medRxiv* 2021; DOI: 10.1101/2021.10.04.21263560.

[100] Park SY Love TMT Perelson AS , et al. Molecular clock of HIV-1 envelope genes under early immune selection. Retrovirology 2016; 13: 38. 27246201 10.1186/s12977-016-0269-6 PMC4888660

[101] Pybus OG Charleston MA Gupta S , et al. The epidemic behavior of the hepatitis c virus. Science 2001; 292: 2323–2325. 11423661 10.1126/science.1058321

[102] Gire SK Goba A Andersen KG , et al. Genomic surveillance elucidates Ebola virus origin and transmission during the 2014 outbreak. Science 2014; 345: 1369–1372. 25214632 10.1126/science.1259657 PMC4431643

[103] Blenkinsop A Pantazis N Kostaki EG , et al. Sources of human immunodeficiency virus infections among men who have sex with men with a migration background: A viral phylogenetic case study in Amsterdam, the Netherlands. J Infect Dis 2024. DOI: 10.1093/infdis/jiae267. PMC11481325 38976562

