# Supplementary material for: Bayesian mixture models for phylogenetic source attribution from consensus sequences and time since infection estimates
Source: Stat Methods Med Res. 2025 Feb 12;34(3):523–44. doi: 10.1177/09622802241309750 (PMC11951470; doi:10.1177/09622802241309750)
Supplement: sj-pdf-1-smm-10.1177_09622802241309750 - Supplemental material for Bayesian mixture models for phylogenetic source attribution from consensus sequences and time since infection estimates [file sj-pdf-1-smm-10.1177_09622802241309750.pdf]

Supplementary Text to

Bayesian mixture models for phylogenetic source attribution from consensus sequences and time since infection estimates: Supplementary Material

Blenkinsop et. al.

Contents

S1 Supplementary Figures **2**

S2 Evolutionary clock model **3**

S3 HSGP random function hyper-parameters and tuning parameters **4**

S4 Epidemic simulation **4**
- S4.1 Model 4
- S4.2 Parameters 6
- S4.3 Patient-level covariates 6

S5 Sensitivity analyses **8**
- S5.1 Age structure of actual and unlinked transmission pairs 8
- S5.2 Amsterdam results with different linear predictors 9
- S5.3 Phylogenetically possible transmission pairs with patristic distances of zero . 10
- S5.4 Alternate background distributions 10

S6 Estimation of age gaps among transmission pairs **12**

S1 Supplementary Figures

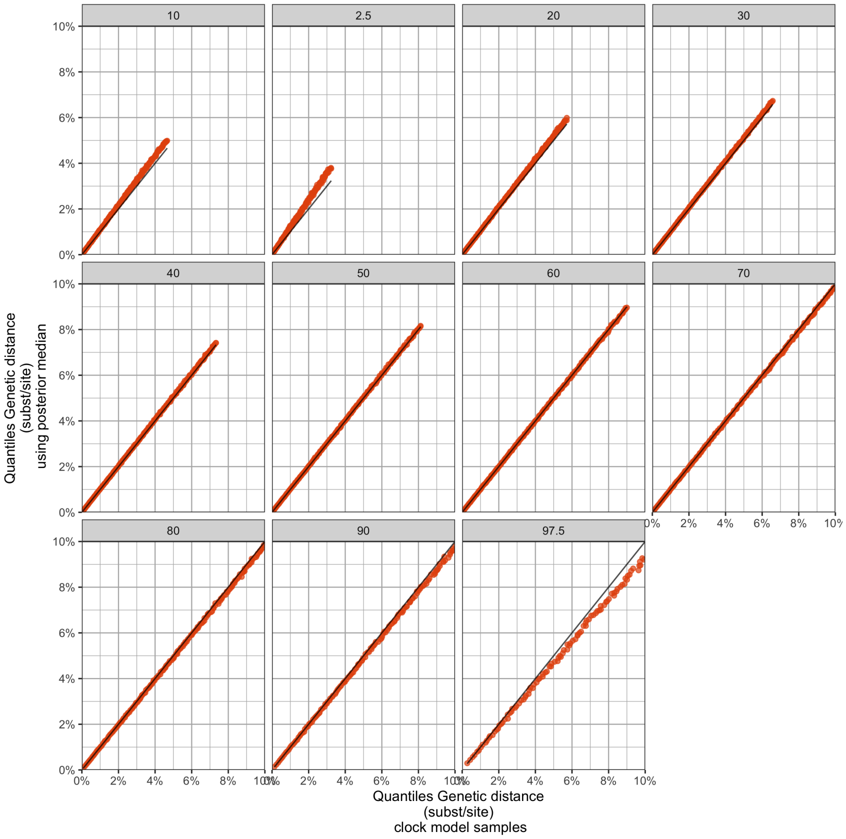

Figure S1: Quantile-Quantile plot for genetic distances predicted by the fitted molecular clock samples and using posterior median estimates to predict distances.

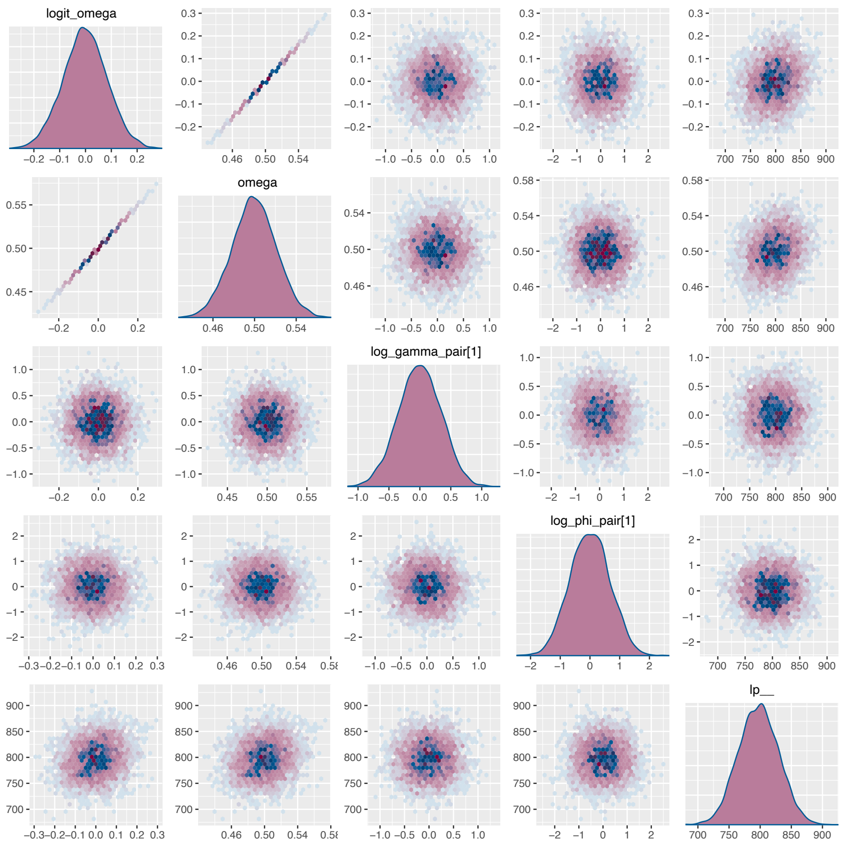

Figure S2: **Pairs plot of the joint posterior density of the vanilla BMM** (13) **parameters in the simulation study.** Omega is the mixing weight, logit_omega is the mixing weight (on the logit scale), log_gamma_pair[1] is the random effect of the evolutionary rate for one example pair in the data, log_phi_pair[1] is the random effect of the dispersion parameter for one example pair in the data.

S2 Evolutionary clock model

The model describing the patristic distances given time elapsed (8) assumes a linear relationship, since in the case of HIV there is substantial evidence to support linear intrahost evolutionary rates in early stages of infection [1, 2, 3, 4]. However the functional form may be flexible in other applications, in which there is evidence of non-linear relationships, and (8) can be adapted accordingly.

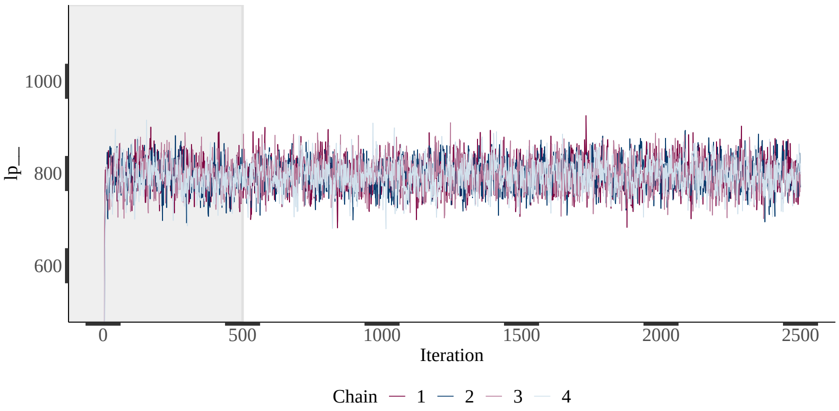

Figure S3: **Trace plot of parameter with the smallest effective sample size for the vanilla BMM in the simulation study.**

S3 HSGP random function hyper-parameters and tuning parameters

In the kernel for the HSGP function (17), hyper-parameters $\boldsymbol{\ell}$ and α are the characteristic length-scale and marginal variance of the kernel, respectively [5]. The length-scale determines the smoothness of the function, and the variance is a scaling factor, determining the deviation of values from their mean. We use the squared exponential covariance function, which has the property of being infinitely differentiable, so ensures ensures good smoothness in the GP function [6].

The HSGP approximates the kernels with additional tuning parameters, m and B. $m = m_1 \times m_2$ are the number of basis functions to approximate each of the kernels, which determines the accuracy of the GP approximation, and are chosen to balance accuracy and computational speed [7]. We chose $m_1 = m_2 = 24$. B is a boundary factor which increases the shifted input domain of $\mathcal{A}$ (centred at zero) to $\Omega = [-L, L]$, defining the domain of the HSGP. Larger values of B improve the the accuracy of the GP approximation, at the cost of computational speed; $B = 1.2$ was chosen using diagnostics to balance these criteria.

S4 Epidemic simulation

S4.1 Model

Transmission pairs were obtained from PopART-IBM [8], a discrete-time agent-based model developed contextually to the HPTN01 trial in Zambia and South Africa [9]. The model is able to simulate demographic processes, as well as HIV transmission dynamics and progres-

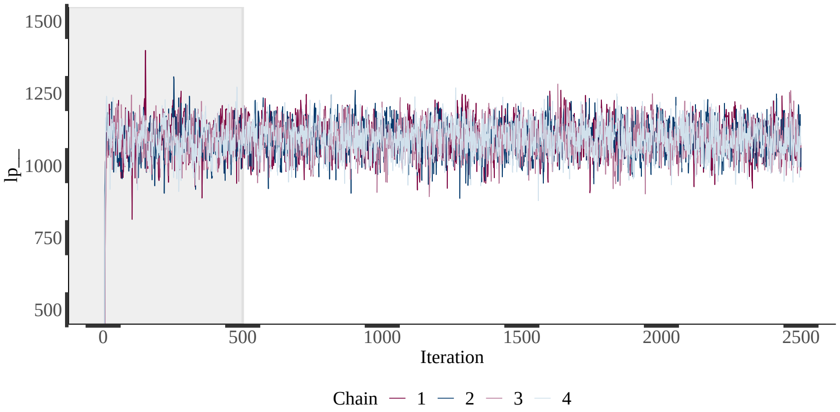

Figure S4: **Trace plot of parameter with the smallest effective sample size for HSGP BMM for Amsterdam MSM.**

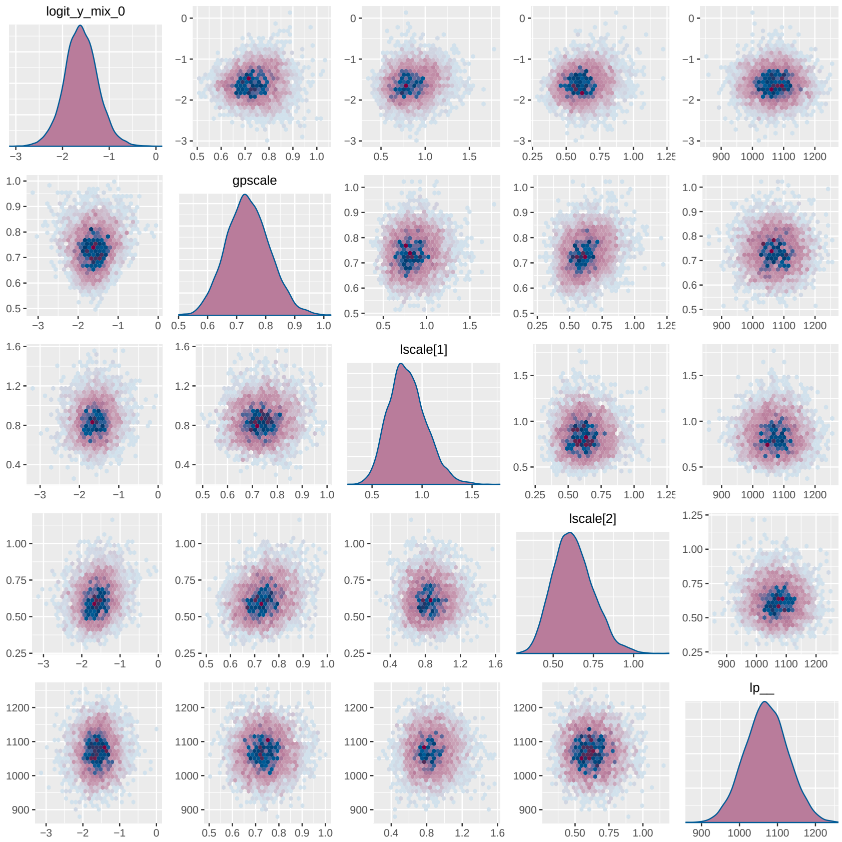

Figure S5: **Pairs plot of the joint posterior density of the HSGP BMM parameters for Amsterdam MSM.**

sion, and interventions at a community level. Interactions between people in the community and the surrounding area (not part of the trial) are also modelled. Only heterosexual partnerships are modelled, and the number of partners individuals may have in their lifetime depends not only on their age and sex, but also on the individual sex activity level, that is determined at birth. The model is informed by means of data collected prior and during the data, including demographic surveys, sexual surveys, data collected from community health care workers who delivered the intervention to households, and from health care facilities. Incidence and prevalence were measured on a cohort of 2000 people, representative of the population aged 18-44, followed up yearly.

PopART-IBM has a number of free parameters, that are calibrated to age-and-sex stratified data on incidence, prevalence, ART uptake, and viral suppression. Calibration is done in each community through Approximate Bayesian Computation algorithms. Transmission trees are obtained by running the model with the best-scoring set of free parameters from one random community, and considering transmission from 2006 onwards, in which at least the infected individual belonged to the community. To each infector-infectee pair we consider metadata including age, sex, set point viral load, cd4 counts of the infector. Simulations start in 1900, and HIV is introduced by infecting a random number of individuals each year between 1965 and 1970.

S4.2 Parameters

The parameters that are calibrated refer broadly to three macro-categories: 1) initialization and HIV introduction; 2) sexual behaviour, including assortativity in partner choice and under-reporting; 3) HIV transmission and progression; 4) Cascade-care and ART effectiveness. The posterior distribution of such parameters is discussed in [8]. Table S1 report the chosen value of each parameter.

The simulation was initiated in 1900 with $N_0 = 4056$, with $32,217$ individuals alive by 1965 when the epidemic was seeded. 148 randomly selected individuals were infected between 1965-1970, and the simulation continued ran until December 2020. Overall, a total of $205,473$ individuals were simulated, with $34,961$ transmission events.

S4.3 Patient-level covariates

Some additional patient covariates were simulated to fit the BMM to the phylogenetically linked transmission pairs.

Time from infection to sequence sampling date, τ_i $(i = 1, \ldots, s)$ were simulated from a Weibull distribution, with shape and scale parameters obtained by fitting a Weibull distribution to time-to-diagnosis estimates from Amsterdam MSM with the R package

Parameter	Value	Description
assortativity	0.660	sexual behaviour assortativity
c_multiplier	3.22	overall underreporting factor in number of partners
breakup_scale_multiplier	1.56	overall base partnership duration
average_annual_hazard	0.092	average annual hazard of transmission per individual
p_HIV_background_testing_pre2006	0.138	background female rate of testing prior to 2006
p_HIV_background_testing	0.219	background female rate of testing after 2006
RR_HIV_background_testing_male	0.924	relative rate of testing for males
p_collect_cd4_test_result_nonpopart	0.949	background probability of entering the cascade care
log_seed_multiplier	1.69	annual number of seeds (1965-1970)
t_start_art_nonpopart	0.444	background time from infection to ART uptake
p_stays_virally_suppressed	0.827	overall probability of remaining virally suppressed after ART
p_stays_virally_suppressed_male	0.989	male probability of remaining virally suppressed after ART
RR_male_to_female_trans	1.538	relative rate of transmission from male to female
initial_low_risk_female	0.456	percentage of low sexual activity individuals - female
initial_low_risk_male	0.487	percentage of low sexual activity individuals - male
initial_med_risk_female	0.826	percentage of average sexual activity individuals - female
initial_med_risk_male	0.758	percentage of average sexual activity individuals - male

Table S1: Free parameters for PopART-IBM.

`fitdistrplus`:

$$\tau_i \sim \text{Weibull}(1.07, 2.89), \tag{S1}$$

as a proxy, assuming that individuals were sequenced shortly after diagnosis. Individuals sequence sampling date, S_i $(i = 1, \ldots, s)$, was therefore defined by,

$$S_i = T_i + \tau_i. \tag{S2}$$

Time elapsed was calculated for all true transmission pairs, and non-transmission pairs using the infection dates and sequence sampling dates using (6). Next, genetic distances for true transmission pairs were simulated by first simulating random effects for each pair,

$$\log \gamma_{ij}^* \sim \mathcal{N}(0, \sigma_\gamma^m) \tag{S3}$$

$$\log \phi_{ij}^* \sim \mathcal{N}(0, \sigma_\phi^m), \tag{S4}$$

where σ_γ^m and σ_ϕ^m denote the medians of the parameters from the molecular clock model. We next simulated the distances with the pair-specific parameters,

$$D_{ij} \sim \text{Gamma}(\alpha_{ij}^*, \beta_{ij}^*), \tag{S5}$$

$$\alpha_{ij}^* = \mu_{ij}\beta_{ij}, \tag{S6}$$

$$\mu_{ij} = (\gamma^m + \gamma_{ij}^*)T_{ij}^e, \tag{S7}$$

$$\beta_{ij}^{*-1} = \phi^m + \phi_{ij}^*, \tag{S8}$$

where γ^m and ϕ^m are the posterior medians from the clock model. The distances for unlinked

pairs were simulated uniformly,

$$D_{ij} \sim \text{Uniform}(0, 0.2). \tag{S9}$$

$$\tag{S10}$$

The ages of the sources, denoted by $x_{ij,1}$, and recipients, $x_{ij,2}$, on the infection date of recipient j, were simulated as follows,

$$x_{ij,1} \sim \text{LogNormal}(\log(30), \log(1.3)^2) \tag{S11}$$

$$x_{ij,2} \sim \text{LogNormal}(\log(x_{ij,1}), \log(1.25)^2), \tag{S12}$$

for $x_{ij,1}, x_{ij,2} \in [16, 75]$. The ages of the sources and recipients for unlinked pairs, were simulated uniformly,

$$x_{ij,1}, x_{ij,2} \sim \text{Uniform}(16, 75). \tag{S13}$$

To explore scenarios with fewer phylogenetically possible pairs per incident case, if c are the average number of possible sources per recipient, $p = 1/c$ are the proportion of total pairs corresponding to true transmission pairs. After formulating all potential pairs and applying exclusion criteria, we randomly sampled non-transmission pairs to achieve $p = (50\%, 40\%, 30\%, 20\%, 10\%, 8\%)$, such that $c = (2, 2.5, 3.3, 5, 10, 12.5)$.

S5 Sensitivity analyses

S5.1 Age structure of actual and unlinked transmission pairs

To explore the impact of how well the age structure separates the actual transmission pairs from the unlinked pairs in the HSGP BMM, we simulated data in which the age structure of the unlinked pairs was more similar to the actual pairs. To disentangle the impact of how well the bivariate ages of sources and recipients informs their mixing probability from the estimated population-level sources, we considered a binary source category.

The ages of the sources, denoted by $x_{ij,1}$, and recipients, $x_{ij,2}$, on the infection date of recipient j, were simulated similarly as in (S11), with larger variance in the ages of the source to reduce the correlation between the ages within pairs,

$$x_{ij,1} \sim \text{LogNormal}(\log(30), \log(1.3)^2) \tag{S14}$$

$$x_{ij,2} \sim \text{LogNormal}(\log(x_{ij,1}), \log(1.8)^2), \tag{S15}$$

for $x_{ij,1}, x_{ij,2} \in [16, 75]$. The ages of the unlinked pairs were simulated uniformly, as before. Figure S6B shows the bivariate ages within linked and unlinked pairs, in comparison to

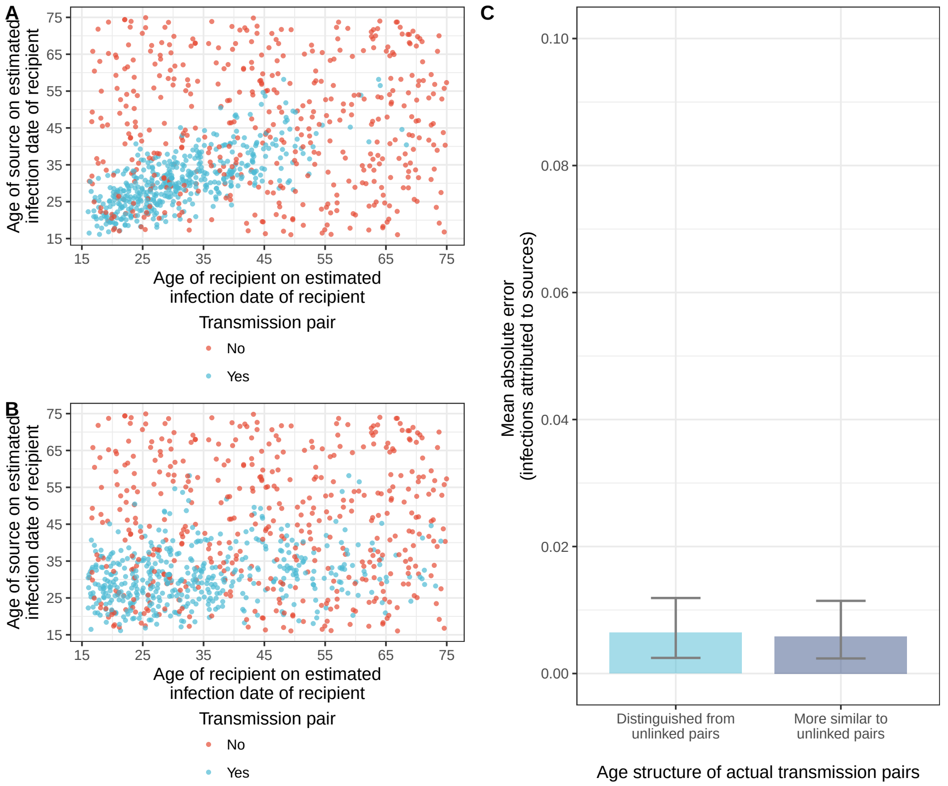

Figure S6: **MAE for binary source category for HSGP model under different age structures of sources and recipients**. (**A**) Structure of the simulated ages of sources and recipients in true transmission pairs and unlinked pairs under data generating procedure given by (S11). (**B**) Structure of the simulated ages of sources and recipients in true transmission pairs and unlinked pairs under data generating procedure given by (S14). (**C**) Posterior median estimates of mean absolute error (MAE) for both scenarios.

Figure S6A from the primary results. The MAE for the estimated sources by five-year age groups was $< 1\%$ in both the model where the age structure of the actual transmission pairs was more distinguished from the unlinked pairs and more similar to the unlinked pairs.

S5.2 Amsterdam results with different linear predictors

We ran simulations to quantify the mean absolute error for a similar configuration of pairs and average number of phylogenetically possible sources per incident case for various different models. Table S2 summarises their mean absolute errors.

We compared the final mixture model fitted to the Amsterdam MSM data to similar models with different linear predictors. Table S3 summarises the expected posterior log density (ELPD) for the four models.

Linear predictor	Mean absolute error
Vanilla (no covariates)	3.5% [3.2-3.8%]
Covariates on grouped ages of sources and recipients	1.1% [0.7-1.5%]
1D random function on age of source	1.3% [0.8-1.8%]
1D random function on age of recipient	2.6% [2.3-2.9%]
1D random functions on both age of source and recipient	0.9% [0.6-1.3%]
2D random function on age of source and recipient	1.2% [0.8-1.6%]

Table S2: Mean absolute error of models with different linear predictors on the mixture probability on the same number of pairs and average number of sources per incident case as the Amsterdam MSM data.

Linear predictor	ELPD
Vanilla (no covariates)	5144.0
Covariates on grouped ages of sources and recipients	5172.4
1D random function on age of source	5178.6
1D random function on age of recipient	5176.6
1D random functions on both age of source and recipient	5164.9
2D random function on age of source and recipient	5180.9

Table S3: Expected posterior log densities for BMM fitted to Amsterdam MSM with different linear predictors on the mixture probability.

S5.3 Phylogenetically possible transmission pairs with patristic distances of zero

Seven Amsterdam MSM estimated to have seroconverted between 2010-2021 had a phylogenetically possible source with an identical sequence, leading to a patristic distance of zero. Since these appeared to be genuine distinct individuals, the patristic distance for these seven pairs was set to one mutation across the length of the alignment (0.077% substitution rate) before fitting the model. We carried out a sensitivity analysis excluding these pairs, which was found to have minimal impact on inferred sources of transmission by age group (Table S4).

S5.4 Alternate background distributions

A uniform background distribution may not be appropriate for some practical applications. Since phylogenetically possible transmission pairs in Amsterdam were formulated from partially observed transmission chains, patristic distances exhibited some degree of clustering. We explored the sensitivity of the inferred transmission flows for Amsterdam to the assumed background distribution.

We first assumed a lognormal background for the distances D_{ij}, parameterized by mean

Age group of source	Estimated transmission sources from age group: Including pairs with zero distances*	Excluding pairs with zero distances
15-29	29.1% [26.5-32%]	29.7% [27-32.8%]
30-39	31.2% [28.6-34%]	30.7% [27.9-33.4%]
40-49	25.2% [22.5-27.9%]	24.9% [22.1-27.6%]
50+	14.4% [11.7-17.1%]	14.8% [12-17.5%]

* Setting their patristic distance to 0.077%

Table S4: Impact on estimated transmission sources among Amsterdam MSM in 2010-2021 from the HSGP BMM by excluding the seven pairs with a patristic distance of zero.

μ^{B}_{ij} for a phylogenetically possible pair with source i and recipient j, and standard deviation σ^{B}, updating the background density to,

$$p(D_{ij}|Z_{ij}=0) = \mathrm{LogNormal}(D_{ij}|\mu^{\mathrm{B}}_{ij}, {\sigma^{\mathrm{B}}}^2) \tag{S16}$$

$$\mu^{\mathrm{B}}_{ij} = \epsilon + \epsilon_{ij} \tag{S17}$$

$$\epsilon \sim \mathcal{N}(-2, 0.5^2) \tag{S18}$$

$$\epsilon_{ij} \sim \mathcal{N}(0, 0.2^2) \tag{S19}$$

$$\sigma^{\mathrm{B}} \sim \mathrm{Exp}(2), \tag{S20}$$

where ϵ is the mean of the lognormal distribution across pairs, and ϵ_{ij} are pair-specific random effects.

We also considered a mixture of K-multivariate normal densities to describe the distribution of patristic distances given time elapsed, parameterized by means $\boldsymbol{\mu}^{\mathrm{B}}_k$, covariance matrices $\boldsymbol{\Sigma}^{\mathrm{B}}_k$ and mixture weights ω^{B}_k for clusters $k = 1, \ldots, K$. To avoid substantial overlap between the signal and background component, we first predicted the patristic distances $\hat{D}_{ij}$ for Amsterdam MSM pairs according to the evolutionary clock model (8) and computed their residuals. For pairs in which $|\frac{D_{ij}-|\hat{D}_{ij}|}{D_{ij}}| > 0.7$, we fitted a 2D Gaussian mixture model (GMM) to the observations (D_{ij}, T^e_{ij}) using the R package `EMCluster`[10], with $K = 5$ clusters. We then used the estimated parameters of the GMM as fixed quantities in the background component of the Bayesian mixture model for source attribution, updating the background density to,

$$p(D_{ij}|T^e_{ij}, Z_{ij}=0) = \sum_{k=1,\ldots,K} \hat{\omega}^{\mathrm{B}}_k \, \mathcal{N}(D_{ij}, T^e_{ij}|\hat{\boldsymbol{\mu}}^{\mathrm{B}}_k, \hat{\boldsymbol{\Sigma}}^{\mathrm{B}}_k). \tag{S21}$$

We next used the following validation test to assess which background distribution was most appropriate. Assuming each incident case can have only one true source, we expect a

model which can correctly identify true transmission pairs to recover a mean posterior mixing weight across pairs, given by $\omega = \text{inv-logit}(\eta_0)$ in (17), which is close to the expected number of transmission events as a proportion of all possible pairs. In our application, given 409 incident cases and 2,824 phylogenetically possible transmission pairs, we expected an average mixing weight of approximately $409/2,824 = 0.14$ (14%). We compared this to the inferred mixing weight from the three models, which was 17% [95% CrI $9-29\%$] assuming a uniform background distribution, 30% [$22-41\%$] with a lognormal background and 53% [$50-52\%$] for a multivariate normal mixture as background. The alternate background distributions therefore led to an over-estimation of the mixing proportion (i.e. true transmission pairs). The implication of unlinked pairs being incorrectly attributed to the signal component, is that signal from the true sources is diluted by non-sources, and the recovered transmission flows from each age group are more similar to the composition of ages among all possible pairs. Thus, specifying background distributions may have a considerable impact on mixture model source attribution analyses.

This validation approach suggests the two alternative candidates for the background density were not suitable for our application. Figure S7 describes the differences in source attribution between the three models with different background densities. Overall, we find the two unsuitable models attributed transmissions to age groups more in line with the population-level age structure among all phylogenetically possible sources.

S6 Estimation of age gaps among transmission pairs

To obtain the age differences between sources and recipients we define,

$$Z_{d,b}|\boldsymbol{X} = \sum_{i:a-b} \sum_{j\in b} \rho_{ij}|\boldsymbol{X}, \tag{S22}$$

where a and b are one-year age bands. We then aggregate over age groups of the recipient, $\tilde{b}$,

$$Z_{d\tilde{b}} = \sum_{b\in\tilde{b}} Z_{db}. \tag{S23}$$

We then define the flows from different one-year age gaps to age group $\tilde{b}$ by,

$$\delta_{d\tilde{b}} = Z_{d\tilde{b}} \Big/ \Big(\sum_{d} Z_{d\tilde{b}}\Big). \tag{S24}$$

We then obtain summary quantiles (25%, 50%, 75%) and minimum and maximum values for each recipient age group, $\tilde{b}$ for each monte carlo sample, then summarise by taking the median for each summary statistic across all samples.

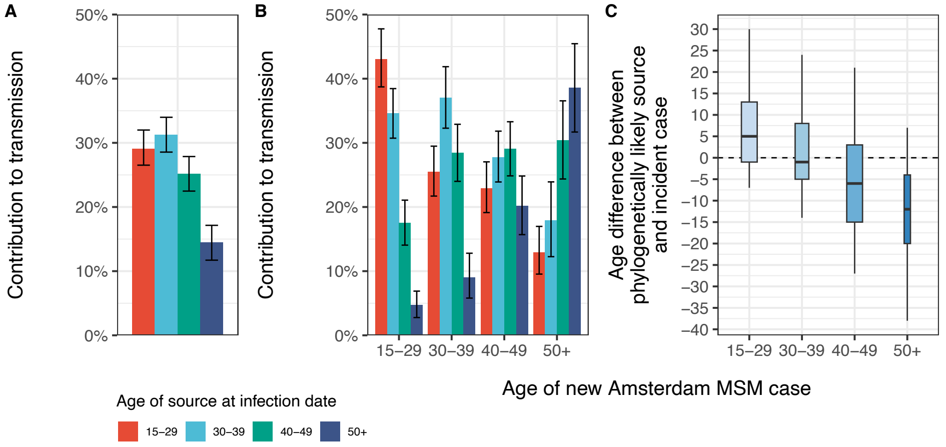

(a) Inferred sources and age gaps assuming Uniform background distribution

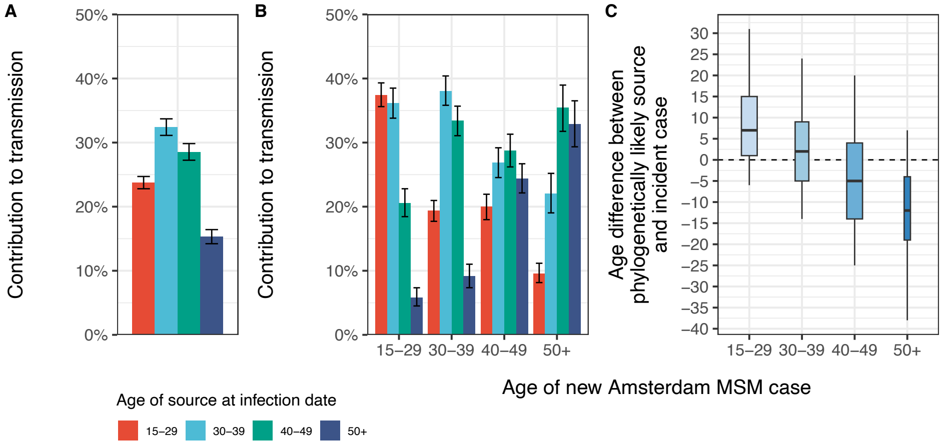

(b) Inferred sources and age gaps assuming lognormal background distribution

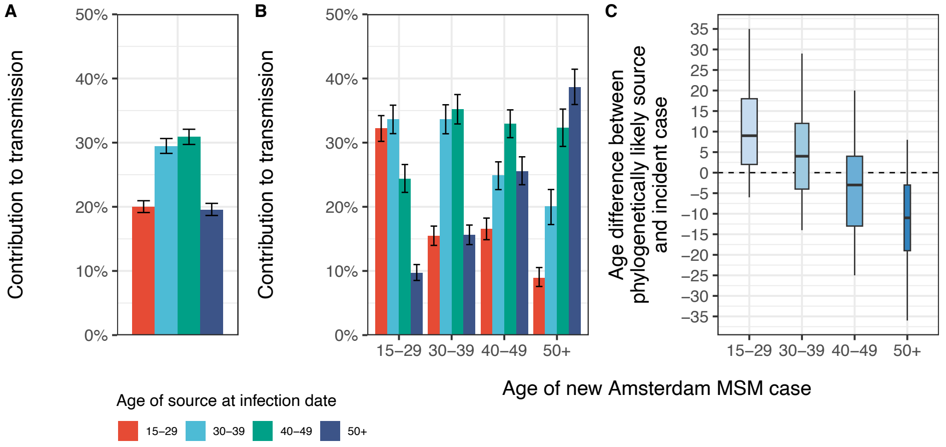

(c) Inferred sources and age gaps assuming multivariate Gaussian mixture background distribution

Figure S7: Inferred source groups and age gaps between sources and recipients, assuming three different background distributions in the Bayesian mixture model.

References

[1] Shankarappa, R. *et al.* Consistent viral evolutionary changes associated with the progression of human immunodeficiency virus type 1 infection. *Journal of Virology* **73**, 10489–10502 (1999). URL http://dx.doi.org/10.1128/jvi.73.12.10489-10502.1999.

[2] Leitner, T. & Albert, J. The molecular clock of hiv-1 unveiled through analysis of a known transmission history. *Proceedings of the National Academy of Sciences* **96**, 10752–10757 (1999).

[3] Zanini, F. *et al.* Population genomics of intrapatient hiv-1 evolution. *eLife* **4** (2015). URL http://dx.doi.org/10.7554/eLife.11282.

[4] Raghwani, J. *et al.* Evolution of hiv-1 within untreated individuals and at the population scale in uganda. *PLOS Pathogens* **14**, e1007167 (2018). URL http://dx.doi.org/10.1371/journal.ppat.1007167.

[5] Solin, A. & Särkkä, S. Hilbert space methods for reduced-rank gaussian process regression. *Statistics and Computing* **30**, 419–446 (2019). URL https://doi.org/10.1007%2Fs11222-019-09886-w.

[6] Rasmussen, C. E. & Williams, C. K. I. *Gaussian Processes for Machine Learning* (MIT Press, 2006).

[7] Riutort-Mayol, G., Bürkner, P.-C., Andersen, M. R., Solin, A. & Vehtari, A. Practical hilbert space approximate bayesian gaussian processes for probabilistic programming. *Statistics and Computing* **33** (2022). URL http://dx.doi.org/10.1007/s11222-022-10167-2.

[8] Pickles, M. *et al.* Popart-ibm, a highly efficient stochastic individual-based simulation model of generalised hiv epidemics developed in the context of the hptn 071 (popart) trial. *PLOS Computational Biology* **17**, 1–21 (2021). URL https://doi.org/10.1371/journal.pcbi.1009301.

[9] Hayes, R. J. *et al.* Effect of universal testing and treatment on HIV incidence — HPTN 071 (PopART). *New England Journal of Medicine* **381**, 207–218 (2019). URL https://doi.org/10.1056/nejmoa1814556.

[10] Chen, W.-C. & Maitra, R. EMCluster: EM algorithm for model-based clustering of finite mixture gaussian distribution (2015). R Package, URL https://cran.r-project.org/package=EMCluster.
